# Resetting our expectations for parasites and their effects on species interactions: a meta‐analysis

**DOI:** 10.1111/ele.14139

**Published:** 2022-11-06

**Authors:** Adam Z. Hasik, Daniela de Angeli Dutra, Jean‐François Doherty, Meghan A. Duffy, Robert Poulin, Adam M. Siepielski

**Affiliations:** ^1^ Department of Biological Sciences University of Arkansas Fayetteville Arkansas USA; ^2^ Jacob Blaustein Center for Scientific Cooperation Ben‐Gurion University of the Negev Midreshet Ben‐Gurion Israel; ^3^ Department of Zoology University of Otago Dunedin New Zealand; ^4^ Department of Zoology University of British Columbia Vancouver British Columbia Canada; ^5^ Department of Ecology and Evolutionary Biology University of Michigan Ann Arbor Michigan USA

**Keywords:** cannibalism, competition, herbivory, host–parasite, meta‐analysis, meta‐analysis of variance, mutualism, parasitism, predation, species interactions

## Abstract

Despite the ubiquitous nature of parasitism, how parasitism alters the outcome of host–species interactions such as competition, mutualism and predation remains unknown. Using a phylogenetically informed meta‐analysis of 154 studies, we examined how the mean and variance in the outcomes of species interactions differed between parasitized and non‐parasitized hosts. Overall, parasitism did not significantly affect the mean or variance of host–species interaction outcomes, nor did the shared evolutionary histories of hosts and parasites have an effect. Instead, there was considerable variation in outcomes, ranging from strongly detrimental to strongly beneficial for infected hosts. Trophically‐transmitted parasites increased the negative effects of predation, parasites increased and decreased the negative effects of interspecific competition for parasitized and non‐parasitized heterospecifics, respectively, and parasites had particularly strong negative effects on host species interactions in freshwater and marine habitats, yet were beneficial in terrestrial environments. Our results illuminate the diverse ways in which parasites modify critical linkages in ecological networks, implying that whether the cumulative effects of parasitism are considered detrimental depends not only on the interactions between hosts and their parasites but also on the many other interactions that hosts experience.

## Graphical abstract

Despite the ubiquitous nature of parasitism, how parasitism alters the outcome of host species interactions such as competition, mutualism, and predation remains unknown. Using a phylogenetically informed meta‐analysis of 154 studies, we examined how the mean and variance in the outcomes of species interactions differed between parasitized and non‐parasitized hosts. Parasitism did not significantly affect the mean or variance of host species interaction outcomes, and our results illuminate the diverse ways in which parasites modify critical linkages in ecological networks, implying that whether the cumulative effects of parasitism are considered detrimental depends not only on the interactions between hosts and their parasites, but also on the many other interactions that hosts experience.

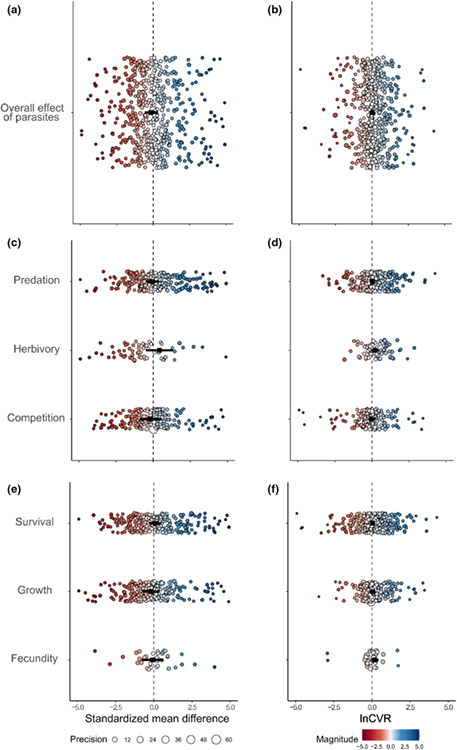

## INTRODUCTION

Most organisms are parasitic and hosts at all trophic levels harbour parasites (Hudson et al., 2002; Price, 1980). For example, Lafferty et al. (2006) showed that more than three‐quarters of all links in a marine food web involved connections with parasites. Although parasites are ubiquitous, their effects are often thought to be minimal, possibly because parasites tend to be aggregated in populations, with a minority of individuals hosting the great majority of parasites (Shaw et al., 1998; Wilson et al., 2002). However, the commonness of parasites may indirectly link them to many other species interactions throughout food webs (Lafferty et al., 2006), or they may have direct effects on other species interactions such as predation and competition (Hatcher et al., 2006, 2012). Despite progress incorporating parasitism into food web ecology (Lafferty et al., 2008; Mougi, 2022), how, why, and to what extent parasitism affects outcomes of species interactions remain unknown. This leaves us with a key gap in our knowledge of how a widespread species interaction modifies other species interactions. Determining effects of parasites on species interactions is therefore a necessary step towards developing a more complete understanding of how communities are structured (Strauss & Irwin, 2004).

The effects of parasite‐mediated changes on species interactions can cascade throughout ecological systems (e.g. Edeline et al., 2016; Liere & Larsen, 2010), not only due to changes in the average outcome of species interactions but also because parasite‐mediated changes can influence the variance in host responses to species interactions. Any effects of parasites on the response variance of their hosts are expected to occur because of the aggregated nature of parasite infection (Shaw et al., 1998; Wilson et al., 2002), differences in individual‐level responses of hosts to infection that result from variation in host resistance/tolerance and/or parasite virulence, and variation among parasites in how strongly they impact interactions (Thomas et al., 2011). Detecting such parasite‐mediated effects on host response variance may therefore reveal potential for natural selection to act on these individual‐level responses to the fitness consequences of parasitism on other species interactions (Strauss & Irwin, 2004), akin to the opportunity for selection (Arnold & Wade, 1984; Crow, 1958).

Parasites are known to moderate the outcome of many species interactions. For example, predators often consume infected prey over uninfected individuals (Duffy et al., 2005; Duffy & Hall, 2008), and parasitized organisms often experience reductions in competitive ability (Grosholz, 1992; Refardt & Ebert, 2012), yet these parasite‐mediated effects are not always negative. Indeed, there are cases in which predators avoid consuming infected prey items (Pourian et al., 2011), being parasitized can make plant resources unpalatable to herbivores (Cipollini & Stiles, 1993), and parasitized individuals have been shown to be superior competitors (Hyder et al., 2013). Examples such as these highlight the possibility that each host–parasite system may have its own, system‐specific response to a given species interaction, with such responses ranging in outcome from negative to positive, resulting in an emergent pattern of no net effect amid a sea of contingencies. Such variation in the outcome of parasite‐mediated effects on host–species interactions makes it crucial to understand if such variation (with no overall effect) is the general trend (Lawton, 1999), or if parasites are as detrimental to host–species interactions as they tend to be to the hosts themselves.

Parasite‐mediated effects on species interactions and fitness components may also vary among the different evolutionary strategies employed by parasites (i.e. parasitoids, parasitic castrators, directly‐transmitted parasites, trophically‐transmitted parasites, vector‐transmitted parasites and micropredators, Poulin, 2011). For example, parasitoids grow within a host and kill it as they emerge into their adult stage. Because they cause considerable damage and usurp resources that could otherwise go towards their host, one may expect them to have greater effects on a host's competitive ability, with no expectation for a consistent effect on predation. In contrast, trophically‐transmitted parasites depend on consumption by a definitive host to complete their life cycle, reducing host survival (Poulin, 2013); thus they are expected to increase negative effects of predation on their intermediate hosts. Indeed, in a recent meta‐analysis on the manipulation of intermediate hosts by their trophically‐transmitted parasites, Fayard et al. (2020) found that these parasites increased predation for infected hosts. However, trophically‐transmitted parasites can also suppress predation for their hosts if the parasite is not yet infective to the definitive host or if the predator is not the definitive host (Médoc & Beisel, 2011). Further, because these parasites manipulate their host to increase transmission to the final host, they may be expected to reduce variation in their host's responses to predation (i.e. all infected hosts respond in a similar manner while non‐infected hosts may exhibit a broader range of responses). While reducing response variance of hosts infected with trophically‐transmitted parasites to predation could be expected, no such effect on response variance would be expected for other parasite evolutionary strategies and species interactions. These examples illustrate that considering parasite evolutionary strategy is important for disentangling potential interactions between parasitism and other species interactions.

Of additional interest is how any effects of parasitism on other species interactions may be influenced by the habitat of hosts and parasites. That is, the effects of parasitism may vary in strength and direction between freshwater, marine and terrestrial habitats, much like the outcomes of predation, competition and mutualisms (Chamberlain et al., 2014). For example, freshwater birds and marine fish have richer parasite communities than terrestrial birds and freshwater fish, respectively (Poulin, 1995). Because they are subjected to infection by a greater diversity of parasites, freshwater birds and marine fish may experience a broader range of effects stemming from species interactions than their terrestrial counterparts. Adaptation to a specific environment by hosts and/or parasites could also affect the strength of parasite‐mediated effects on species interactions. Though they did not study how parasites affected host–species interactions, Cohen et al. (2020) showed in a recent meta‐analysis that freshwater hosts adapted to cold habitats suffered increased parasite prevalence at higher temperatures, yet prevalence in cold‐adapted terrestrial hosts did not change over 40°C gradient. This may have been the result of terrestrial hosts evolving to tolerate a broader range of temperatures (Rohr et al., 2018), highlighting a potential role of the environment in shaping the effects of parasites on species interactions.

The above points illustrate the complex and context‐dependent nature of parasites and their pernicious effects on host organisms, which when combined with their potential for population‐ and community‐level effects (Hatcher et al., 2014; Thieltges et al., 2013), highlight the need for a comprehensive review and analysis of the effects of parasites on species interactions. We simply do not know what the emergent and general effects of parasites are on species interactions hosts experience. Moreover, studies have not traditionally considered parasites in their experimental designs and theoretical frameworks, despite many calls to do so (Cohen et al., 1993; Gehman et al., 2019; Kuris et al., 2008; Marcogliese & Cone, 1997). This is potentially problematic, because if effects of a focal interaction are exaggerated (or reduced) when a host is parasitized, and parasitism is not accounted for, the magnitude of a focal interaction may be over‐ or underestimated. That is, the overall effect of competition or predation may not be entirely because of that interaction – it may instead reflect the added effect of unaccounted parasitism.

The goal of this study was to assess how parasites shape the outcome of species interactions. To achieve this goal, we used a meta‐analysis to quantitatively summarise how infection from a diverse range of parasites affects (via mean or variance responses) species interactions to address five questions. First, we asked how parasitism affects the outcome of species interactions. Second, we determined what effect parasites have on the fitness components (growth, survival and fecundity) of their hosts. Third, we asked if parasite evolutionary strategy affected the influence of parasitism on species interactions. Fourth, we asked if the nature of the infected host (i.e. parasitized prey vs. parasitized predator) affected the strength of the effect of parasitism on species interactions. Fifth, we asked if the host–parasite habitat affected the strength of the effect of parasitism on species interactions. Overall, we found that parasites had no net effect on host–species interaction mean responses or response variance, yet infection could increase or decrease the negative effects of species interactions depending on the nature of the infected host, parasite evolutionary strategy or habitat.

## METHODS

### Literature search and classifications

We performed a systematic literature search for studies investigating how parasitism affects species interactions. Figure S1 visualises the study selection process with a PRISMA flow chart (Salameh et al., 2020). We searched for relevant articles on ISI Web of Science up to 28 July 2022, using the following topic search queries in multiple, separate searches: “parasit* OR fung* OR bact* OR virus OR pathogen OR infection OR disease AND (predat* OR compet* OR mutual* OR herbiv* OR scaveng* OR omniv* OR cannibal* OR intraguild predat* OR detritiv* OR apparent competition)”, refining our search to only include papers in the “ecology”, “zoology”, “evolutionary biology” and “biology” categories. We also included the term “NOT human” to exclude non‐relevant studies on humans for our *virus* search.

We applied the following criteria to select relevant articles: first, the study had to measure the impact of parasitism on a species interaction: interspecific competition, intraspecific competition, mutualism, predation, herbivory, cannibalism, ammensalism or commensalism. Studies that measured a proxy for predation, such as parasitoids consuming host biomass, were discarded. Studies included all life stages, experimental and observational studies, in addition to both field‐ and laboratory‐based studies.

Second, we defined parasitic organisms as parasites, parasitoids, parasitic castrators and micropredators (e.g. fleas and mosquitoes), because we were interested in the effect that a parasitic organism has on species interactions that its host engaged in, though we did not include brood parasites or social parasites. While these organisms acquire host resources, they are not true parasites that acquire resources directly from living on or in hosts. We also excluded studies that analysed effects of endosymbiotic bacteria *Wolbachia* and mycorrhizal fungi on host organisms, as both *Wolbachia* (Zug & Hammerstein, 2015) and mycorrhizal fungi (Hoeksema et al., 2010) are defined as mutualists that can become “parasitic” and thus are not true parasites, as well as excluding studies that used heat‐killed parasites, as this limited effects of the parasite as seen when allowed to normally infect its host.

Third, because we were interested in effects of parasites on species interactions, not how those effects vary with parasite intensity (i.e. number of parasites per infected host), studies had to measure differences in a metric of performance (e.g. percent survival) for a species interaction between non‐parasitized and parasitized groups. Studies in which the non‐parasitized group only had their parasite load reduced (e.g. Hoi et al., 2018) or those where authors could not detect low levels of infection (e.g. Zylberberg et al., 2015) were discarded. In the *Discussion* we return to the issue of parasite load and how it may affect species interactions.

Our literature search returned a total of 63,045 articles, of which 62,940 were excluded for not meeting inclusion criteria, leaving us with 104 studies. We also included a total of 14 studies identified from three previous meta‐analyses (Fayard et al., 2020; Fernandez‐Conradi et al., 2018; Flick et al., 2016), and searched for papers that cited those already included in our database, garnering a further 36 studies. Thus, we had a total of 154 studies.

If studies did not provide the necessary data to calculate effect sizes needed for our meta‐analysis (means, standard deviations and sample sizes), we contacted authors directly (*n* = 22 studies). If data could not be acquired by these means, we used figures from the studies to extract relevant information (*n* = 85 studies) using ImageJ ver. 1.53a (Schneider et al., 2012).

### Moderators and effect size calculations

For each study, we extracted the following moderators: (1) species interaction, (2) fitness component, (3) parasite evolutionary strategy, (4) agent parasitized and (5) habitat. For the moderator species interaction, studies were grouped by the species interaction investigated: competition, mutualism, predation, herbivory and cannibalism. For the fitness component moderator, studies were grouped by the fitness component (fecundity, individual growth and survival) impacted by parasites in a given species interaction, allowing us to relate host performance to fitness (Arnold, 1983). We were only able to extract fecundity fitness component effect sizes from predation and competition studies. For the moderator parasite evolutionary strategy, studies were grouped by parasite classification (parasitoid, castrator, directly‐transmitted parasite, trophically‐transmitted parasite, vector‐transmitted parasite or micropredator). For the moderator agent parasitized, we focused on predation, competition and herbivory studies, classifying the agent parasitized as either predator or prey (for predation studies), which competitor was measured (parasitized or non‐parasitized for competition studies) and which type of competition was measured (inter‐ or intraspecific), or herbivore or plant (for herbivory studies). For the habitat moderator, studies were grouped by the habitat (freshwater, marine or terrestrial) in which a given host–parasite pair was tested in (for field studies) or sourced from (for laboratory studies).

To examine the effects of parasitism, we used two effect size measures: one to compare differences in host mean responses between parasitized and non‐parasitized groups, and one to compare differences in the variances of host responses between these groups. For mean responses, we used Hedge's *g*, or the standardised mean difference (SMD) – the difference between two groups in units of standard deviations (Hedges, 1981). Hedge's *g* was calculated by subtracting the mean of the non‐parasitized group from the mean of the parasitized group, and then dividing the difference by the pooled standard deviation. Studies often used different scales when measuring host performance: larger or more positive values could indicate a more beneficial mean outcome (higher survival) or a more detrimental mean outcome (greater mortality) for hosts. As such, we converted means, when necessary, by multiplying them by negative one, ensuring all were on the same scale. We report Hedge's *g* as *Ɵ* [95% confidence interval], with negative SMD values representing a detrimental effect of parasites on hosts (e.g. reduced survival), while positive SMD values represent an advantageous effect of parasites on hosts (e.g. increased survival).

To compare differences in variances between parasitized and non‐parasitized groups, we used the natural logarithm of the ratio between the coefficients of variation from two groups (lnCVR, Nakagawa et al., 2015). To standardise, we used absolute values of mean responses for parasitized and non‐parasitized groups, because several studies reported negative mean response values. We report lnCVR as *Ɵ* [95% confidence interval] and calculated it such that positive values indicate how many parasites increase variance in host responses, and negative values indicate a decrease in host response variance.

We used the R package *metafor* (Viechtbauer, 2010) to calculate all effect sizes. Hedge's *g* effect sizes were calculated using the “SMDH” measure option in the escalc function, as the variances of the parasitized and non‐parasitized groups were heteroscedastic (Bonett, 2009), while lnCVR effect sizes were calculated using the “CVR” measure option in escalc.

### Statistical analysis

We constructed separate multi‐level mixed‐effect models (Nakagawa & Santos, 2012; Viechtbauer, 2010) for each of our core questions. Categorical moderators (species interaction, fitness component, parasite evolutionary strategy, agent parasitized, habitat) were included as fixed effects in separate, univariate models. Additionally, in cases where we had sufficient sample size we also constructed multivariate models to test interaction effects (see *Results*).

Because most studies (*n* = 103) had multiple effect sizes, each effect size was not independent. Therefore, to take into account the correlated structure of this dataset, we nested each effect size within study and included both terms as random effects (Nakagawa & Santos, 2012). We used a restricted maximum‐likelihood estimator to calculate the amount of residual heterogeneity (*τ* ^2^) among effect sizes (Viechtbauer, 2005). We also calculated *I* ^*2*^, which estimates the amount of heterogeneity relative to the total amount of variance in the observed effects or outcomes. All models were built with the rma.mv function in *metafor*, which weights each effect size by the inverse of its sampling variance (Gurevitch & Hedges, 1999).

### Incorporating phylogeny

One additional source of non‐independence is the shared evolutionary histories of hosts and parasites (Chamberlain et al., 2012). Closely‐related hosts could exhibit similar responses to infection for a given interaction, or two closely‐related parasites could have similar effects on a host's species interactions. To account for these sources of non‐independence, we included host and parasite phylogeny as random effects in our models (details in Supplementary Material Section 2). Interactions between hosts and parasites are not likely to be dependent solely on either hosts or parasites, but instead, relate to the shared evolutionary history of both organisms (Hadfield et al., 2014). We therefore also included a random effect of the interaction of the host and parasite phylogenies by calculating tensor products of the correlation matrices (Hoeksema et al., 2018; Lynch, 1991), in addition to accounting for non‐phylogenetic random effects of host and parasite species (Supplementary Material Section 2).

Multiple effect sizes in our dataset (*n* = 71 effect sizes from *n* = 15 studies) were extracted from studies investigating viruses, which do not have a resolved position on the Tree of Life, and *n* = 3 effect sizes were extracted from *n* = 1 study that involved an unclassified parasite. As such, we could not examine every study in our complete dataset while controlling for phylogeny. We therefore conducted three sets of analyses on two separate datasets. The first analysis controlled for phylogeny and excluded viruses/unclassified parasites, the second analysis did not control for phylogeny and excluded viruses/unclassified parasites, while the third analysis did not control for phylogeny and included viruses/unclassified parasites. The first dataset included only studies for which we could control for the phylogeny of hosts and parasites (*n* = 508 effect sizes from *n* = 138 studies), while the second dataset included viruses/unclassified parasites (*n* = 582 effect sizes from *n* = 154 studies). Differences between the two sets of analyses conducted on the first dataset would indicate that shared evolutionary histories influenced the effects of parasites on host–species interactions, while differences between the analyses conducted on the first and second dataset would indicate that removing viruses/unclassified parasites influenced the overall estimated effect of parasites.

We did not find evidence for publication bias towards large effects of parasites (negative or positive) on mean responses or response variance using funnel plots (Figure S2). Trim‐and‐fill analyses revealed that no effect sizes were missing for any of the analyses of either effect size, indicating there was no asymmetry in the data.

We also examined for potentially influential outliers with Cook's distance *d* (Cook, 1977). Any effect size with values of *d* greater than three times the mean was considered an outlier (see Supplementary Material Section 2 for summary information), and we ran our analyses again without these outliers.

## RESULTS

### Overview of studies in the database and overall effects of parasitism

**FIGURE 1:**
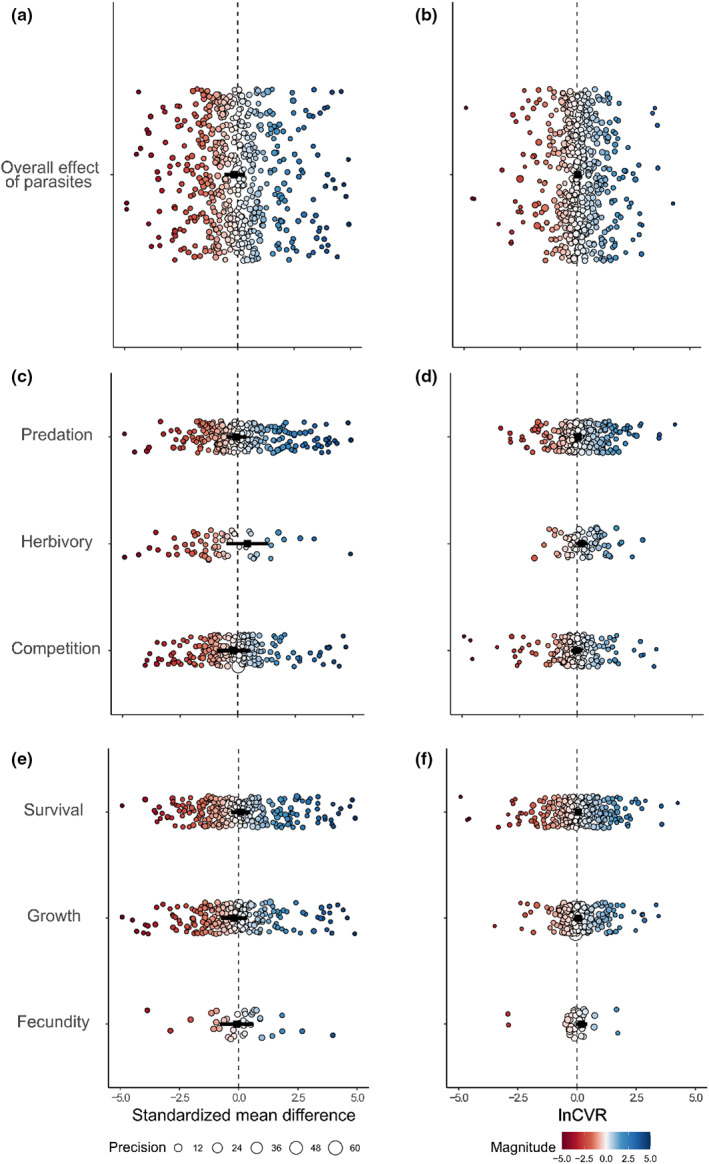
Overall effects of parasites on species interaction mean responses (a) and response variance (b), effects of parasites on competition, herbivory, and predation mean responses (c) and response variance (d), and effects of parasites on fecundity, growth, and survival mean responses (e) and response variance (f). (a), (c) and (e) only show SMD effect sizes from −5 to 5 (Figure S5 shows the full range). Negative SMD values represent a detrimental effect of parasites on hosts (i.e. reduced survival), while positive values represent an advantageous effect of parasites on hosts (i.e. increased survival). Large points in each panel denote the estimated effects of parasites on mean fitness and fitness variation, respectively. Smaller points in each panel are individual effect sizes, colour (hotter – more negative, cooler – more beneficial) denotes magnitude and sign of effect sizes, diameter denotes precision of estimates (1/SE), and error bars represent 95% CI's.

Most studies focused on effects of parasites on predation (*n* = 93, Figure S3) and effects of parasites on invertebrate (*n* = 89) and vertebrate (*n* = 33) animal hosts (Figure S3a). The majority of parasites were either fungi, arthropods or platyhelminths (*n* = 47, 29 and 26 studies respectively, Figure S3b). Only seven of the 154 studies measured the effects of parasites on mutualisms, and two studies measured the effects of parasites on cannibalism, therefore we removed these nine studies from further analyses beyond determining the overall effects of parasites (e.g. Figure 1a,b).

Because there were no qualitative differences controlling for phylogeny and excluding viruses/unclassified parasites and including non‐phylogenetic random effects terms (Figure S4a,b), controlling for phylogeny and excluding viruses/unclassified parasites (Figure S4c,d), not controlling for phylogeny and excluding viruses/unclassified parasites (Figure S4e,f), or removing outliers and including viruses/unclassified parasites (Figure S4g,h), we focus our analyses on the most comprehensive dataset ‐ including viruses/unclassified parasites and not controlling for phylogeny. Importantly, we found no difference in the effects of parasites on species interactions between field‐ and laboratory‐based studies, nor when investigators used previously‐infected hosts or controlled infection levels in the experiments (Supplementary Material Section 2). Only two observational studies investigated the effects of parasites on species interactions, therefore we did not investigate differences in parasite‐mediated effects between experimental and observational studies.

Overall, across all studies, our analyses revealed no significant effect for Hedge's *g* (Figure 1a), indicating that on average parasites have neither a consistent detrimental nor beneficial effect on the outcome of all species interactions when viewed collectively. Additionally, there was no significant effect for lnCVR (Figure 1b), indicating parasites neither consistently increased nor decreased response variance. Instead, for both effect size measures there was considerable variation in these outcomes, and accordingly, all models had a high degree of heterogeneity associated with their measured effects (Table 1).

**TABLE 1:** Summary statistics for the heterogeneity index (*I* ^2^), which is a measure of the total variability in the effect size estimates which can be attributed to heterogeneity among true effects

Analysis	Heterogeneity index (*I* ^2^)
Models of mean responses	
Controlling for phylogeny, no viruses	*I* ^2^_*total*_ = 99.41% *I* ^2^_*among studies*_ = 42.67% *I* ^2^_*within studies*_ = 7.47% *I* ^2^_*host phylogeny*_ = 49.27% *I* ^2^_*parasite phylogeny*_ = 1.52 e‐09% *I* ^2^_*host phylogeny x parasite phylogeny*_ = 7.63 e‐06%
Without controlling for phylogeny, no viruses	*I* ^2^_*total*_ = 99.11% *I* ^2^_*among studies*_ = 87.44% *I* ^2^_*within studies*_ = 11.67%
Without controlling for phylogeny, with viruses	*I* ^2^_*total*_ = 98.96% *I* ^2^_*among studies*_ = 85.06% *I* ^2^_*within studies*_ = 13.90%
Models of responses variance	
Controlling for phylogeny, no viruses	*I* ^2^_*total*_ = 91.87% *I* ^2^_*among studies*_ = 33.40% *I* ^2^_*within studies*_ = 16.94% *I* ^2^_*host phylogeny*_ = 41.53% *I* ^2^_*parasite phylogeny*_ = 5.53 e‐08% *I* ^2^_*host phylogeny x parasite phylogeny*_ = 2.25 e‐07%
Without controlling for phylogeny, no viruses	*I* ^2^_*total*_ = 88.90% *I* ^2^_*among studies*_ = 65.94% *I* ^2^_*within studies*_ = 22.91%
Without controlling for phylogeny, with viruses	*I* ^2^_*total*_ = 87.93% *I* ^2^_*among studies*_ = 64.93% *I* ^2^_*within studies*_ = 23.01%

*Note*: Each analysis is a measure of the overall effects of parasites on host mean responses or response variance, *I* ^2^_*total*_ represents the heterogeneity of the full model, while the other *I* ^2^ values represent the amount of heterogeneity explained by the random effects in each model.

### Do parasites affect the outcome of species interactions?

When considering only competition, herbivory and predation, we found that the effects of parasites on the mean responses of each species interaction were highly variable, ranging from strongly deleterious to strongly beneficial, with no significant overall effects (Figure 1c). Likewise, the effects of parasites on the variance of responses in species interactions were similarly variable, and none of these relationships were significant (Figure 1d).

### Do parasites affect the fitness components of hosts?

The effects of parasites on mean responses had variable effects on growth, fecundity and survival, but none of these effects were statistically significant (Figure 1e). The effects of parasites on response variance were similarly variable, and none were statistically significant (Figure 1f).

### Do the effects of parasites on species interactions vary with parasite evolutionary strategy?

**FIGURE 2:**
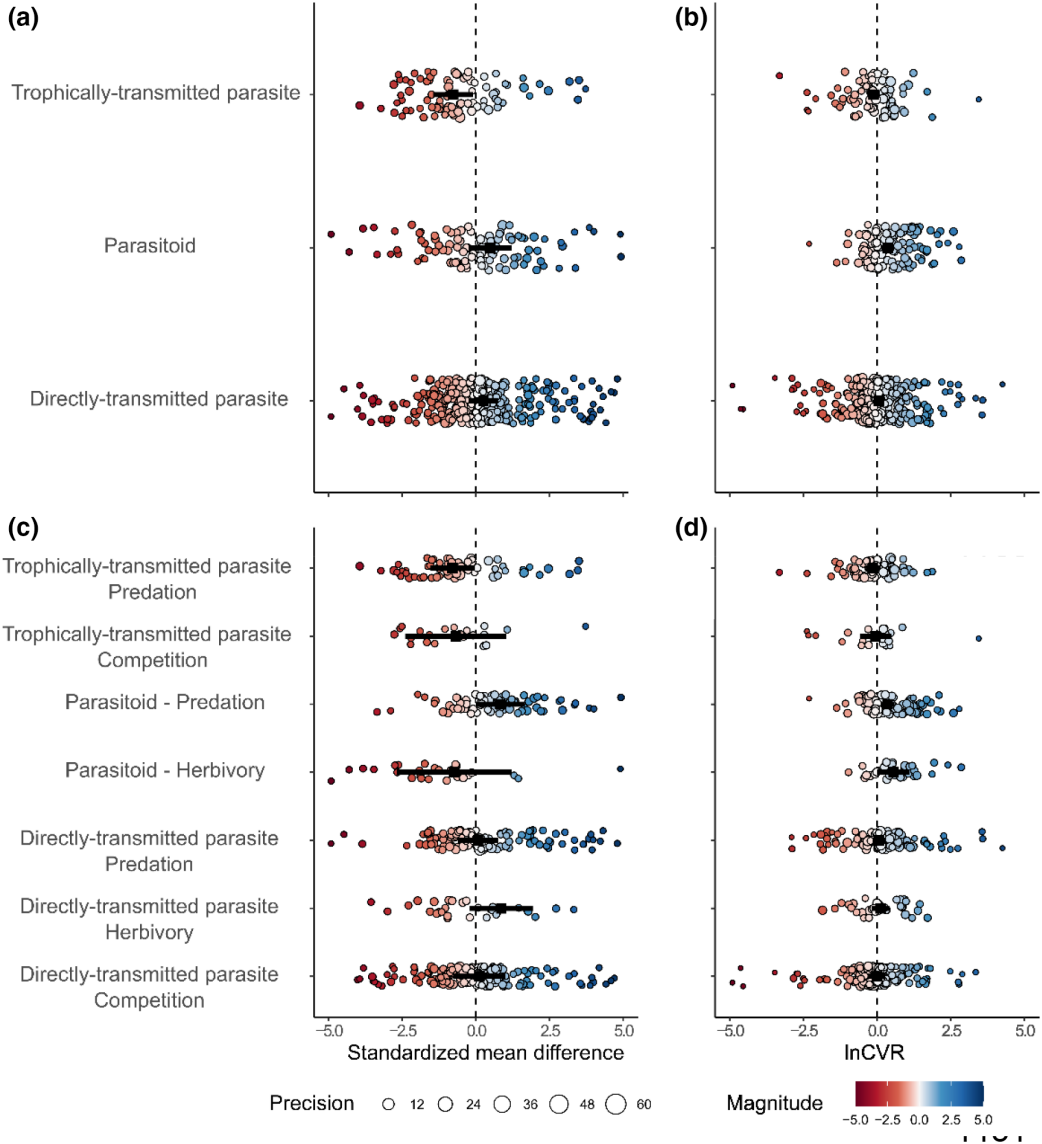
Effects of parasites on mean responses (a) and response variance (b), divided by parasite evolutionary strategy. (c) and (d) show the effects of parasites on mean responses and response variance, respectively, divided by parasite evolutionary strategy and species interaction. (a) and (c) only show SMD effect sizes from −5 to 5 (Figure S5 shows the full range). Negative SMD values represent a detrimental effect of parasites on hosts (i.e. reduced survival), while positive values represent an advantageous effect of parasites on hosts (i.e. increased survival). Large points in each panel denote the estimated effects of parasites on mean fitness and fitness variation, respectively. Smaller points in each panel are individual effect sizes, colour (hotter – more negative, cooler – more beneficial) denotes magnitude and sign of effect sizes, diameter denotes precision of estimates (1/SE), and error bars represent 95% CI's.

Due to the limited number of effect sizes extracted from studies of parasitic castrators (*n* = 9 effect sizes from *n* = 3 studies) and micropredators (*n* = 4 effect sizes from *n* = 2 studies), we only analysed effects of directly‐transmitted parasites, parasitoids and trophically‐transmitted parasites, with the latter measured exclusively in intermediate hosts. We found that trophically‐transmitted parasites had a large, negative effect on host mean responses (*Ө*_*trophic*_ = −0.78 [−1.45, −0.10]), though there were no significant effects of directly‐transmitted parasites or parasitoids on host mean responses (Figure 2a). For effects of parasites on host response variance, we found that parasitoids moderately increased response variance (*Ө*_*parasitoid*_ = 0.36 [0.15, 0.57]), though neither directly‐ nor trophically‐transmitted parasites had a significant effect on response variance (Figure 2b).

We did not find a significant interaction between the effects of parasite evolutionary strategy and species interaction type on host mean response (Wald‐type tests of mean response model coefficients: *QM* = 11.86, *df* = 7, *p* = 0.11, pseudo‐*R* ^2^ = 2.38). Trophically‐transmitted parasites markedly increased the negative effects of predation (*Ө*_*trophic‐predation*_ = −0.80 [−1.56, −0.05]), whereas parasitoids tended to reduce the negative effects of predation on average (though note the 95% CI overlaps with 0, Figure 2c). That is, hosts infected with trophically‐transmitted parasites were substantially more likely to be consumed, and those infected with parasitoids were somewhat less likely to suffer predation, though the latter was not significant. We also did not find a significant interaction between parasite evolutionary strategy and species interaction type for response variance (Wald‐type tests of response variance model coefficients: *QM* = 14.31, *df* = 7, *p* = 0.05, pseudo‐*R* ^2^ = 4.61). Parasitoids moderately increased response variance in both herbivory (*Ө*_*parasitoid‐ herbivory*_ = 0.56 [0.004, 1.11]) and predation (*Ө*_*parasitoid‐ predation*_ = 0.35 [0.11, 0.59]), though the other parasite evolutionary strategies had no effect on the other interaction types (Figure 2d).

### Do the effects of parasites depend on the agent parasitized, or the nature of competition?

**FIGURE 3:**
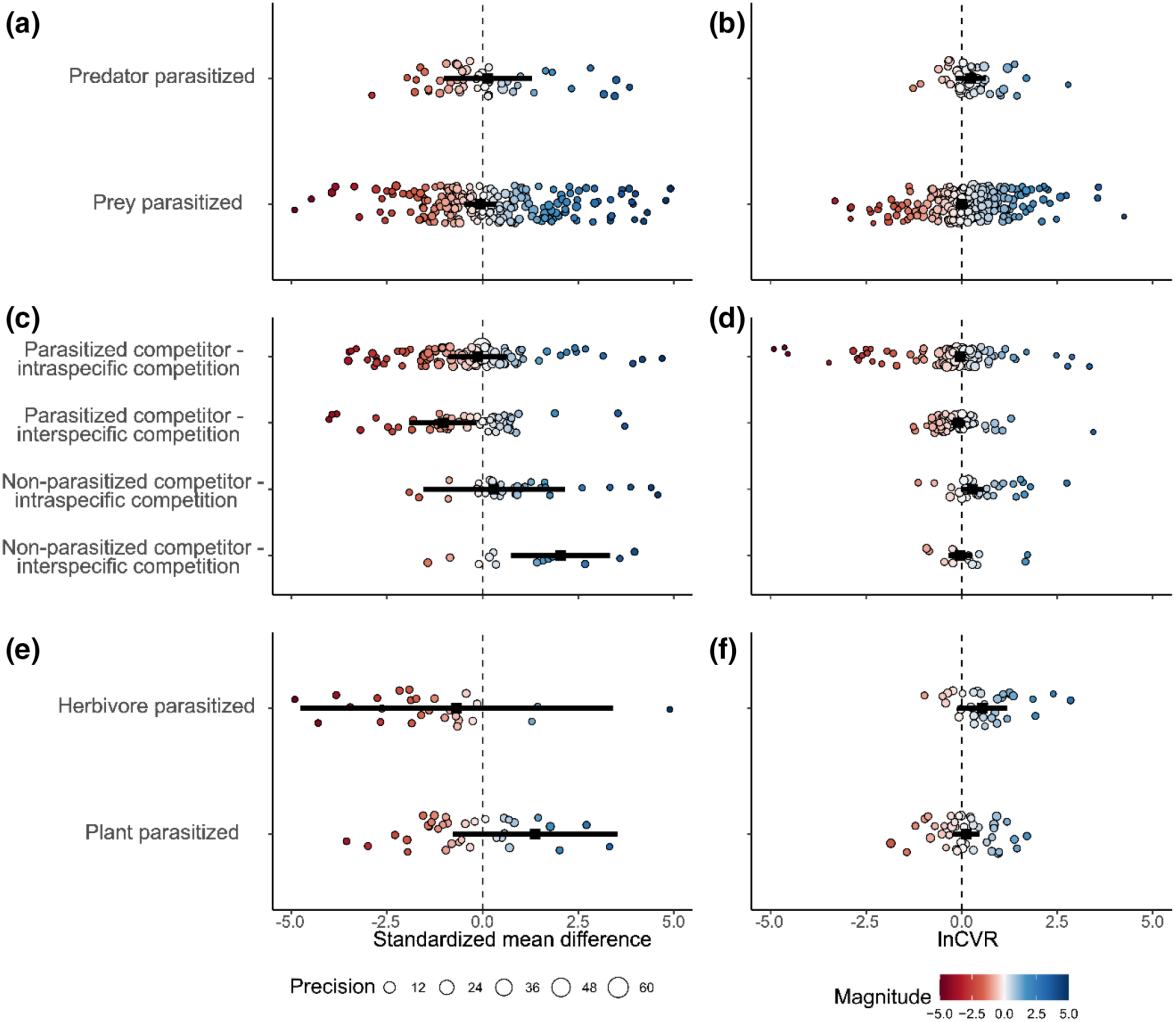
Effects of parasites on predation mean responses (a) and response variance (b), inter‐ and intraspecific competition mean responses (c) and response variance (d), in addition to the effects of parasites on herbivory mean responses (e) and response variance (f), divided by agent parasitized. (a), (c) and (e) only show SMD effect sizes from −5 to 5 (Figure S5 shows the full range). Negative SMD values represent a detrimental effect of parasites on hosts (i.e. predators are less efficient, prey are consumed more), while positive values represent an advantageous effect of parasites on hosts (i.e. predators are more efficient, prey are consumed less). Large points in each panel denote the estimated effects of parasites on mean fitness and fitness variation, respectively. Smaller points in each panel are individual effect sizes, colour (hotter – more negative, cooler – more beneficial) denotes magnitude and sign of effect sizes, diameter denotes precision of estimates (1/SE), and error bars represent 95% CI's.

For the effects of parasites on predation, we found no difference in effects on mean responses when either prey or predators were parasitized (Figure 3a). This indicates that infected predators were neither more nor less effective in consuming prey, and that infected prey were, overall, neither more nor less likely to be consumed. Likewise, there were no effects of parasites on response variance when either prey or predators were parasitized (Figure 3b).

We found a significant interaction between competition type and agent parasitized (Wald‐type tests of mean response model coefficients: *QM* = 17.27, *df* = 4, *p* = 0.002, pseudo‐*R* ^2^ = 17.06). Parasites reduced the competitive ability of parasitized hosts when competing with heterospecifics (*Ө*_*parasitized ‐ interspecific*_ = −1.04 [−1.92, −0.16]), yet had no effect on parasitized hosts when competing with conspecifics (Figure 3c). Non‐parasitized competitors benefitted from competing with parasitized heterospecifics (*Ө*_*non‐parasitized ‐ interspecific*_ = 2.04 [0.74, 3.34]) but there was no effect when non‐parasitized competitors were competing with parasitized conspecifics (Figure 3c). We did not find a significant interaction between competition type and agent parasitized for response variance (Wald‐type tests of response variance model coefficients: *QM* = 4.59, *df* = 4, *p* = 0.33, pseudo‐*R* ^2^ = 7.59), nor were there significant effects of parasites on variance in competitive abilities of parasitized or non‐parasitized competitors (Figure 3d).

Investigating the effects of parasites on herbivory, we found that there was no difference in the effects on mean responses when either the plant or the herbivore was parasitized (Figure 3e). Likewise, there were no effects of parasites on response variance when either the plant or the herbivore was parasitized (Figure 3f).

### Do the effects of parasites vary among habitats?

**FIGURE 4:**
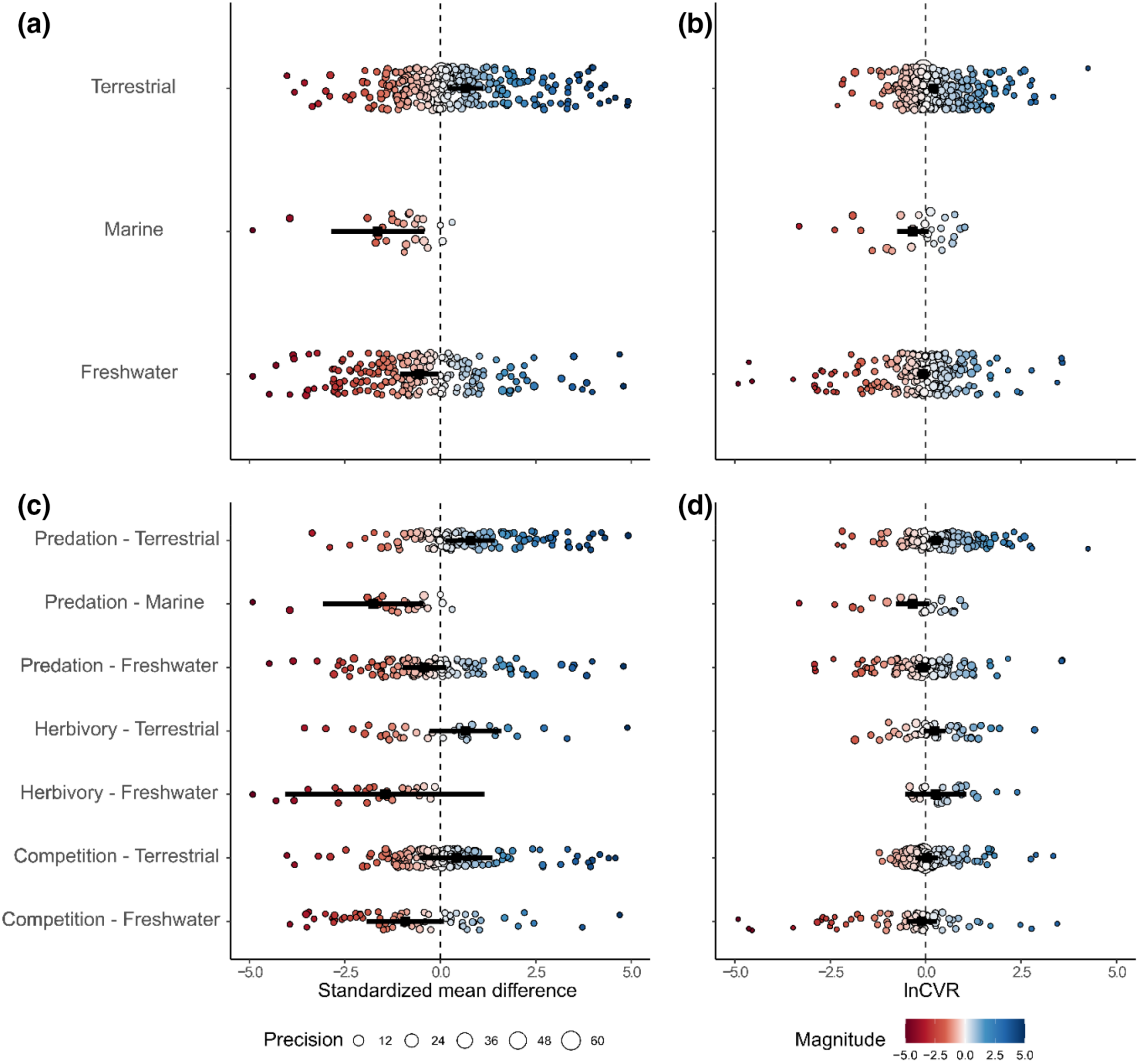
Effects of parasites on mean responses (a) and response variance (b) in various habitats, in addition to the effects of parasites in various habitats on mean responses (c) and response variance (d), divided by species interaction. (a) and (c) only show SMD effect sizes from −5 to 5 (Figure S5 shows the full range). Negative SMD values represent a detrimental effect of parasites on hosts (i.e. parasitized hosts benefit less/suffer more negative effects from a given species interaction in a given habitat), while positive values represent an advantageous effect of parasites on hosts (i.e. parasitized hosts benefit more/suffer fewer negative effects from a given species interaction in a given habitat). Large points in each panel denote the estimated effects of parasites on mean fitness and fitness variation, respectively. Smaller points in each panel are individual effect sizes, colour (hotter – more negative, cooler – more beneficial) denotes magnitude and sign of effect sizes, diameter denotes precision of estimates (1/SE), and error bars represent 95% CI's.

We found that parasites had beneficial effects on host mean responses to species interactions in terrestrial habitats (*Ө*_*terrestrial*_ = 0.66 [0.20, 1.12]), negative effects in marine habitats (*Ө*_*marine*_ = −1.64 [−2.87, −0.42]) and moderately negative effects in freshwater habitats (*Ө*_*freshwater*_ = −0.56 [−1.06, −0.05], Figure 4a). We also found that parasites slightly increased response variance in terrestrial habitats (*Ө*_*terrestrial*_ = 0.20 [0.06, 0.35]), with no effect in freshwater or marine habitats (Figure 4b).

To further investigate the effects of parasites in varying habitats, we asked if parasites from these three habitats had differential effects on competition, herbivory and predation. We excluded the effects of parasites on herbivory in marine habitats due to limited sample sizes (*n* = 3 effect sizes from *n* = 1 study) and found a significant interaction between habitat and species interaction type (Wald‐type tests of mean response model coefficients: *QM* = 20.82, *df* = 7, *p* = 0.004, pseudo‐*R* ^2^ = 10.48). Parasites had large, negative effects on predation in marine habitats (*Ө*_*marine – predation*_ = −1.75 [−3.07, −0.43]), yet were beneficial for predation in terrestrial habitats (*Ө*_*terrestrial – predation*_ = 0.78 [0.14, 1.43], Figure 4c), with no significant effects on other species interactions in other habitats (Figure 4c). There was no significant interaction between habitat and species interaction type for response variance (Wald‐type tests of response variance model coefficients: *QM* = 12.76, *df* = 7, *p* = 0.08, pseudo‐*R* ^2^ = 2.74). Parasites moderately increased response variance for predation in terrestrial habitats (*Ө*_*terrestrial – predation*_ = 0.27 [0.06, 0.47], Figure 4d), but did not affect response variance of other species interactions in other habitats (Figure 4d).

## DISCUSSION

Overall, our analysis revealed that parasite‐mediated effects covered the entire range of host responses from overwhelmingly beneficial to overwhelmingly detrimental – there was no consistent effect of parasites on either the average or variance in the outcomes of species interactions of their hosts. Both effects were similarly variable among all species interaction types and host fitness components. Controlling for the shared evolutionary histories of hosts and parasites and excluding viruses/unclassified parasites also did not qualitatively change effects of parasites on the outcome of species interactions. Differences among the effects of parasite evolutionary strategies only emerged when considering the effects of parasite evolutionary strategy on predation of their hosts, and differences among the effects of parasites on species interactions were only apparent when considering parasitized and non‐parasitized heterospecific competitors. Parasites had negative and positive effects in aquatic and terrestrial habitats, respectively, yet these effects varied when considering interaction type, namely predation. Below, we place our results in the broader context of understanding how parasitism influences the outcome of species interactions.

### Do parasites affect the outcome of species interactions?

Across studies, parasites had variable effects on how well hosts performed in species interactions – below we consider each interaction in turn, focusing on predation and competition as they make up most studies. Interestingly, there was no consistent effect of parasites on interactions with predators. Parasitized hosts are often thought to be disproportionately consumed by predators due to changes in their behaviour (Otti et al., 2012) or reductions in their defences against predators (Slattery et al., 2013). Parasites can also reduce predator‐avoidance behaviours in their hosts, with some that cause their hosts to become attracted to predator cues, resulting in a homogenised response to predators in parasitized prey individuals, facilitating the transmission of the parasites to their final hosts (Benesh et al., 2008). Importantly, the lack of an overall trend points to a more varied effect of parasites on predation that is not merely due to a collection of negative and null effects, but instead due to large numbers of both positive and negative effects.

Like predation, parasites tended to slightly reduce the competitive abilities of their hosts on average and had variable effects ranging from beneficial to deleterious, yet we found consistent differences among inter‐ and intraspecific competition. Specifically, parasites had limited effects on intraspecific competition, but increased the competitive ability of non‐parasitized individuals when competing with parasitized heterospecifics and decreased the ability of parasitized individuals to compete with non‐parasitized heterospecifics. For example, parasitized guppies (*Poecilia reticulata*) experienced reduced growth when competing with non‐parasitized killifish (*Rivulus hartii*, Pérez‐Jvostov et al., 2016), yet the aboveground biomass of non‐parasitized native *Coix lacryma‐jobi* plants increased when competing with the invasive and parasitized *Mikania micrantha* (Li et al., 2019). This change in the outcome of interspecific competition suggests that parasitism affects interspecific competition in a way that could impact local coexistence between competitors. Stable local species coexistence can occur when species experience stronger population regulation in response to conspecific than heterospecific competitors. This stabilising effect (Chesson 2000) can therefore be undermined if interspecific competition intensifies relative to intraspecific competition. Unfortunately, studies did not report how the strength of intra relative to interspecific competition was altered under parasitism. However, the limited overall effects on intraspecific competition and finding that parasites increase the negative effects of interspecific competition for parasitized hosts, while non‐parasitized competitors benefit more from interspecific competition sets the stage for parasite‐mediated competitor extirpation. Indeed, theory predicts that coexistence is impossible when the negative effects of a species interaction strengthen interspecific competition more than intraspecific competition, even when fitness differences between the competitors are negligible (Singh & Baruah, 2020). Our finding that parasites may destabilise coexistence via their effects on interspecific competition offers an exciting and important avenue for future research.

### The nuanced effects of parasites on host–species interactions

These results imply that effects of parasites on predation and competition require careful consideration of the context‐dependent and system‐specific nature of individual host–parasite associations. Indeed, these system‐specific effects may have been why we did not find significant differences among our phylogenetically informed and naïve analyses. Each host–parasite association may involve such specific nuances that even closely‐related parasites have completely different effects on their hosts, or a given host may have a radically different relationship with a given parasite than its sister species. Our analyses also do not consider the potential for what is likely substantial population‐level variation in the outcomes of parasitism; exploring this more fully is an important future goal.

More generally, though, the considerable variation in the outcome of species interactions when simultaneously contending with parasitism demands additional study to understand the underlying mechanisms whereby parasites shift from being costly to beneficial. Is this merely unpredictable noise, or are there instead generalisations that can be made? This may be a sign that there are many cases where the strict textbook definition of “parasite” (i.e. something that negatively impacts host fitness) does not apply. The study of mutualisms too has been undergoing a similar shift in thinking and most species interactions are notoriously context specific (Mushegian & Ebert, 2016; Scott et al., 2022). The large range of effects in our results may imply that an organism that is a parasite in one environment might be a commensal or mutualist in another, at least when considered in light of other species interactions. For example, when infected with microsporidians, *D. dentifera* suffered reduced fecundity when virulent, obligate‐killer parasites were not common and resources were scarce due to the detrimental effects of the microsporidians (Rogalski et al., 2021). However, when these virulent, obligate‐killer parasites were present the microsporidian‐infected *D. dentifera* were more fecund than their uninfected counterparts, as the virulent parasites could not penetrate the gut wall (Rogalski et al., 2021). Findings such as this example, combined with our result that parasites can be either beneficial or detrimental, highlight the importance of context and nuance for host–parasite interactions, as found in other species interactions (Holland, 2014).

Regardless of the underlying mechanisms, a key implication of our results is that the effects of species interactions are often likely tempered by the additional effects of parasitism. Given how common parasitism is, this means that the effects of predation, competition and herbivory in many cases may not reflect the true magnitude of the effect of predators, competitors or herbivores alone. Instead, they may reflect the combined action of how parasitism is affecting another interaction. These results are consistent with previous work that has illustrated the potential for myriad outcomes to emerge when one interaction is considered in light of other species interactions. For example, predation can enhance, reduce or have no effect on competition (Chase et al., 2002). Moreover, feedbacks between interactions are common; for example predation not only interacts with and affects competition (Chase et al., 2002), but the strength of competition also depends on the intensity of predation (Gurevitch et al., 2000; Siepielski et al., 2020). Yet, because the cascading effects of parasitism are rarely accounted for (Cohen et al., 1993; Gehman et al., 2019; Kuris et al., 2008; Marcogliese & Cone, 1997), we run the risk of over‐ or underestimating any direct negative effects of a focal species interaction.

### Do parasites affect the fitness components of hosts?

The effects of parasites on fitness components were also quite variable, which could be due to system‐specific relationships among predators, prey (hosts) and parasites. For example, when infected with an apicomplexan parasite (*Eimeria vermiformis*), mice (*Mus musculus*) preferred predator odours over neutral odours (Kavaliers & Colwell, 1995), a change in behaviour that reduces host survival, yet increases transmission of the parasite to its final feline host. However, in an onion thrip host (*Thrips tabaci*) fungal parasite (*Metarhizium anisopliae*) system the survival of parasitized thrips was significantly higher than that of non‐parasitized thrips (Pourian et al., 2011). Unlike the negative effects of parasites on the survival of mice, this apparent benefit of parasitism for the thrips is because the predators (flower bugs, *Orius albidipennis*) actively avoided feeding on parasitized thrips. For the variable effects on fecundity, the nature of the agent parasitized may provide clarity. When competing with non‐parasitized conspecifics, infected *A. thaliana* were less fecund (Creissen et al., 2016), but non‐parasitized soybean plants (*Glycine max*) were more fecund when competing with parasitized velvetleaf (*Abutilon theophrasti*). Beyond the host's interactions with other organisms, the effects of the parasite on the host itself provide further illumination, as Dick et al. (2010) hypothesised that the combined metabolic demands of the parasite and host likely caused the increased foraging rate of parasitized *Gammarus pulex*, though whether this increased foraging resulted in a greater benefit for the host or parasite requires further testing. In contrast, Parris and Cornelius (2004) found that toads (*Bufo fowleri*) infected with chytrid (*Batrachochytrium dendrobatidis*) exhibited reduced growth compared to non‐parasitized grey treefrogs (*Hyla chrysoscelis*), which was likely due to chytrid‐mediated reduction in feeding efficiency (Fellers et al., 2001). The disparate nature of these results implies that understanding the effects of parasites on host fitness components also requires careful consideration of the nature of the species interaction, in addition to the relationship of the parasite with its host and any potential predators.

### Do the effects of parasites on species interactions vary with parasite evolutionary strategy?

We found that trophically‐transmitted parasites were consistently associated with hosts experiencing negative outcomes to interspecific interactions, confirming the results of Fayard et al. (2020). However, trophically‐transmitted parasites did not decrease response variance for their intermediate hosts. Such increased predation for hosts makes sense, as parasites must be consumed by definitive hosts to complete their lifecycle, yet natural selection should favour trophically‐transmitted parasites that also generate a decrease in response variance to further facilitate transmission. That is, because hosts infected with trophically‐transmitted parasites must be consumed by a definitive host (Poulin, 2011), and many hosts are manipulated by these parasites to exhibit very specific behaviour (Poulin, 2013), one might expect for these hosts to display similar responses to predation. Such a lack of an effect, even for these notorious manipulators, further speaks to how contingency among parasites, hosts and their predators rules species interactions.

### Do the effects of parasites vary among habitats?

We found that the effects of parasites on the mean outcome of species interactions of their hosts were negative in freshwater and marine habitats, but beneficial in terrestrial habitats, with parasites increasing response variance in terrestrial habitats as a whole and for interactions with predators. Further investigation revealed that parasites in marine habitats increased the negative effects of predation on response means, while terrestrial parasites reduced mean effects of predation. These contrasting results could be due to the way that the parasite impacts the relationship between the predator and prey (host). For example, the unclassified parasite in Slattery et al. (2013) increased the predation of its coral host because infection likely weakened both structural and chemical defences, facilitating predation by butterfly fishes (*Chaetodon* spp.) compared to uninfected coral. In contrast, many terrestrial predation studies included in our database investigated the effects of parasitoids on predation of their hosts (e.g. Fu et al., 2017), and predators avoided feeding on infected prey because the infected prey are a lower‐quality resource (Bilu & Coll, 2009). Though not significant, we also found that parasites tended to reduce the mean response of freshwater hosts for herbivory and competition, yet benefitted infected hosts in terrestrial habitats on average. Like the effects of parasites on host fitness components, this variation is due to system‐specific differences. For example, freshwater *D. dentifera* infected with fungi (*Metschnikowia bicuspidate)* greatly reduce their foraging rates (Penczykowski et al., 2022), yet terrestrial roses (*Rosa hybrida*) “benefitted” from infection by the fungus *Botrytis cinerea* because herbivores preferred to consume uninfected plant tissue (Mouttet et al., 2011). Similar “benefits” in terrestrial habitats can be due to associations with other organisms. For example, Albornoz et al. (2016) found that infection with fungi (*Phytophthora* spp.) improved the ability of plant hosts (*Calothamnus calothamnus*) to compete for phosphorous. When uninfected, their competitors were much more efficient at acquiring phosphorous, but infected *C. calothamnus* utilised their associations with ectomycorrhizal fungi to increase their competitive ability, as their competitors lacked ectomycorrhizal fungi and as such could not acquire sufficient resources when infected (Albornoz et al., 2016).

### Limitations and future directions

While this study contributes a novel synthesis of the impact of parasites on interspecific interactions, it nonetheless has several limitations that themselves point to future directions. We focused our analysis on the effects of parasite occurrence, but not on how these effects may increase with infection intensity, as this was rarely reported. Despite the lack of studies, considering parasite intensity is likely to be an important factor for better understanding parasite‐mediated effects on the means, and especially the variance in the outcome of species interactions. Indeed, Risely et al. (2017) found in a meta‐analysis of the effects of parasitism on host migration that increasing intensity was associated with decreases in host movement, phenology and survival. Further, in a meta‐analysis of host body condition and parasite infection, Sánchez et al. (2018) showed that body condition decreased as infection intensity increased. Future work on the effects of parasites on species interactions should therefore incorporate infection intensity. The latter is potentially important, because most parasitized individuals have few parasites (Wilson et al., 2002), and any effects of parasites that are detected may be due to over‐representation by heavily infected individuals.

Despite an exhaustive literature search reviewing >63,000 studies, we only found two studies on cannibalism, and seven on mutualisms. A better understanding of how parasitism affects mutualisms is clearly warranted given that virtually all species are engaged in some form of mutualistic interaction. Indeed, we have limited understanding of how infection between the players involved in mutualisms may be differentially impacted by parasitism. For example, how do the outcomes of a plant‐pollinator mutualism change, when either the plant or pollinator (or both) are parasitized? If differences in mutualism success (e.g. a plant being successfully pollinated and its pollinator being rewarded) are contingent on parasitism status, this may help to account for unexplained variation in the outcome of mutualistic interactions in nature (Chamberlain et al., 2014). Hatcher et al. (2006) called for future models of competition to incorporate infection status of the competing organisms, and our results not only highlight the importance of incorporating infection status into competition studies (especially interspecific competition) but also show that extending this more broadly to all species interactions could be fruitful for capturing more realistic effects of species interactions in communities.

In addition, more work is needed to understand indirect, trait‐mediated effects (Werner & Peacor, 1993) of parasites on species interactions. Despite collecting over 580 effect sizes on the effects of parasites on species interactions, only 54 of those effect sizes were extracted from some indirect effect of parasites on species interactions. Moreover, we found only one study that specifically investigated the indirect effects of parasites on another species interaction (but see Mouritsen & Haun, 2008 and Wood et al., 2007 for similar studies that did not meet our inclusion criteria). Bernot and Lamberti (2008) found that snail communities with increasing prevalences of a trematode parasite consumed more algae, reducing algal biomass and altering periphyton community composition. Future studies should also investigate a wider variety of hosts, as we found a disproportionate representation of animal hosts in studies of parasite effects on species interactions, most of which were invertebrates, with only two studies using a fungal host. As such, we were unable to directly investigate effects of parasites among different host taxa, beyond that captured in our models showing limited effect of phylogeny.

Finally, future studies should focus on understanding the mechanisms behind why infections with parasites can shift from costly to beneficial. Disentangling these varying effects will ultimately require experimental approaches. Researchers should also test for varying effects across environmental gradients, as the effect of parasites on species interactions not only varies among habitat types (this study), but may also vary across abiotic and biotic gradients. This effect of environmental context, combined with the limited number of studies extracted from field experiments (*n* = 42 effect sizes from *n* = 19 field studies), emphasises the necessity of exploring parasite‐mediated effects on species interactions in natural, field settings.

## CONCLUSIONS

Overall, our meta‐analysis revealed remarkably varied effects of parasites on species interactions, emphasising the lack of general and overarching patterns in ecology (Lawton, 1999). These results have implications for measures of species interactions, such that any study that fails to account for the effects of parasitism on species interactions is likely to over‐ or underestimate the magnitude of the effects of interactions such as competition, predation or herbivory. Previous work on synthesising multiple interaction effects has found that not only can one species interaction impact another (Chase et al., 2002) but also that the strength of species interactions are often dependent on other species interactions (Gurevitch et al., 2000). Our results add to this work, as well as that incorporating the effects of parasites into community ecology (Flick et al., 2016; Hatcher et al., 2006; Lafferty et al., 2008; Poulin, 2013; Tompkins et al., 2011). Our study highlights multiple gaps in our understanding of the effects of parasites on species interactions, especially cannibalism, indirect effects and mutualisms.

Parasitism is “defined” by the negative impact that one organism has on another. However, our review has shown that this idea can be overturned when also considering how parasitism may affect the outcome of interactions that a host has with other organisms, which may ultimately determine host fitness. This result implies that it may be time to reset our expectations for the effects of parasites (at least in the context of their effects on other species interactions). No species exists in an ecological vacuum (Thompson, 2005), and studies investigating these relationships are needed to understand the role of parasites within species interaction networks. The interactions making up the structure of food webs are complex, and accounting for this complexity and potential for feedbacks to emerge among species interactions is necessary to develop a more complete understanding of how communities are structured.

## AUTHOR CONTRIBUTIONS

AZH and AMS conceived the study. AZH, MAD, RP and AMS designed the study. AZH, DaAD and JFD collected data, and AZH and AMS performed modelling work and analysed data. AZH wrote the first draft of the manuscript, and all authors contributed substantially to revisions.

### PEER REVIEW

The peer review history for this article is available at https://publons.com/publon/10.1111/ele.14139.

## Supporting information


Data S1

## Data Availability

All data and code associated with this study are deposited on Data Dryad: https://doi.org/10.5061/dryad.wdbrv15sb.
