# Supplementary material for: Resetting our expectations for parasites and their effects on species interactions: a meta‐analysis
Source: Ecol Lett. 2022 Nov 6;26(1):184–99. doi: 10.1111/ele.14139 (PMC10099232; doi:10.1111/ele.14139)
Supplement: Supplementary file 1 — Data S1 [file ELE-26-184-s001.docx]

**Supplemental information for:**

**Resetting our expectations for parasites and their effects on species interactions: a meta-analysis**

This file contains:

Section 1: Figures S1-S5, Table S1-S2

Section 2: Additional details on phylogenetic tree construction, data summary and analyses, and a list of papers included in our database

**Section 1**

**
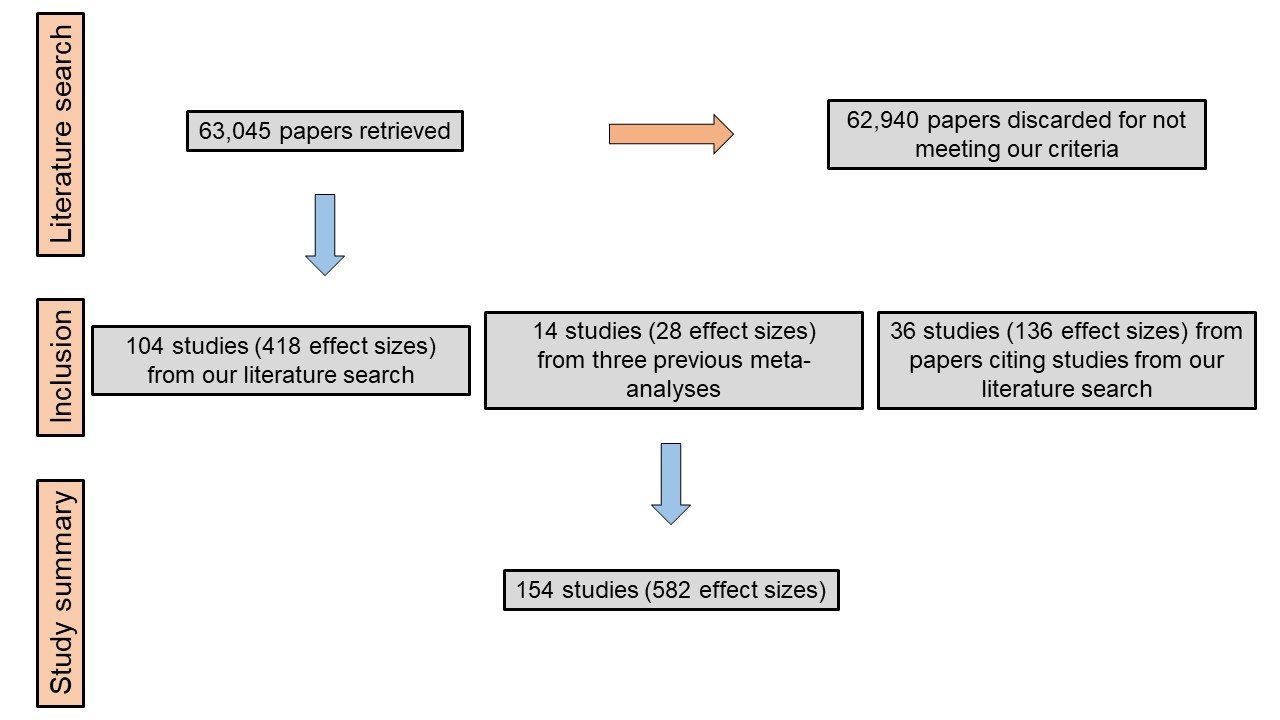
Figure S1.** – PRISMA flow chart of the study inclusion process.


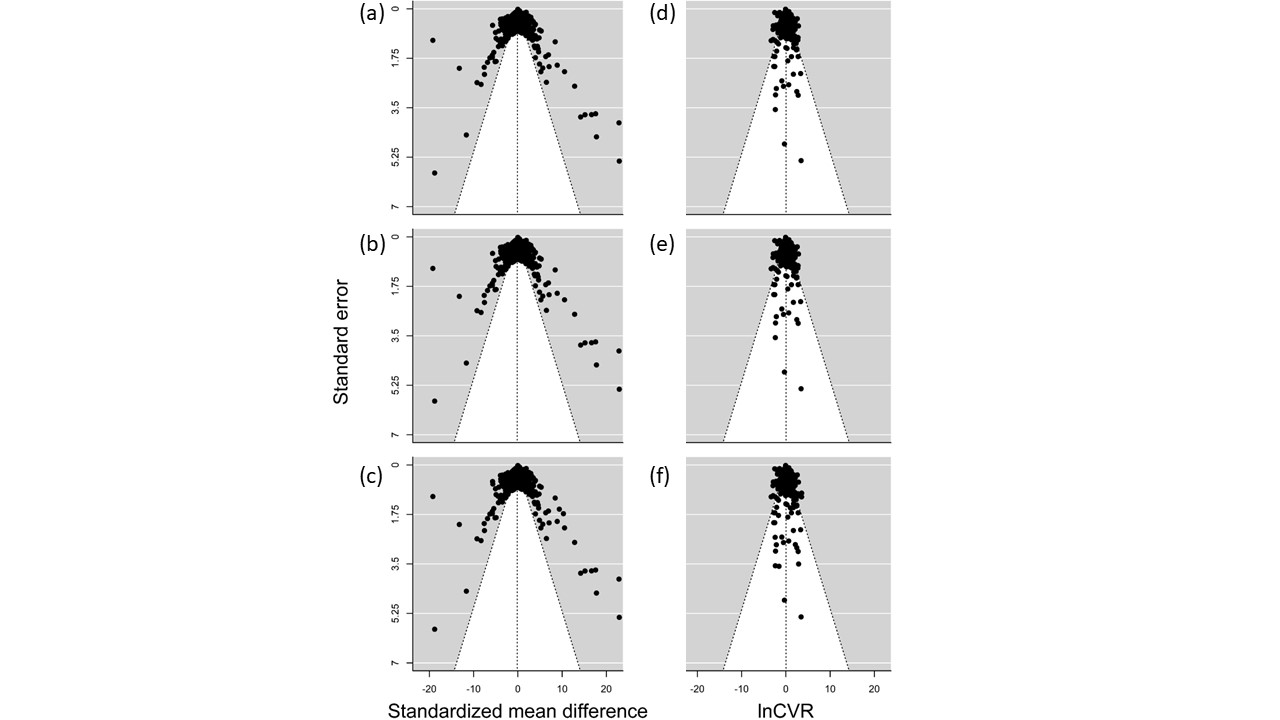


**Figure S2.** – Funnel plots for models of mean responses that controlled for phylogeny (a), did not control for phylogeny (b), or did not control for phylogeny and contained viruses/unclassified parasites (c), as well as funnel plots for models of response variance that controlled for phylogeny (d), did not control for phylogeny (e), or did not control for phylogeny and contained viruses/unclassified parasites (f).

**
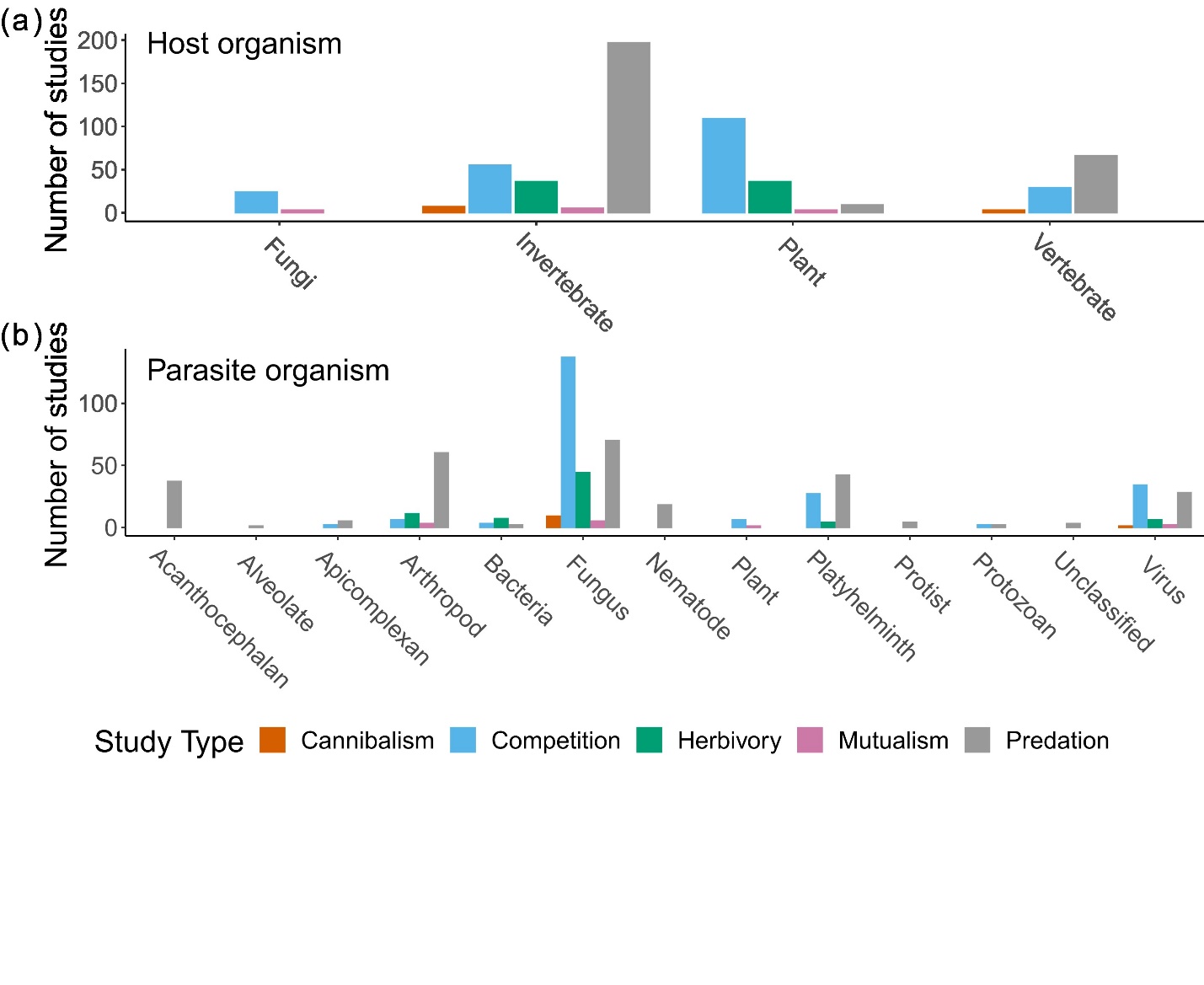
**

**Figure S3.** – Counts of the number of studies analyzing each species interaction included in our database separated by host (a) or parasite organism (b).

**
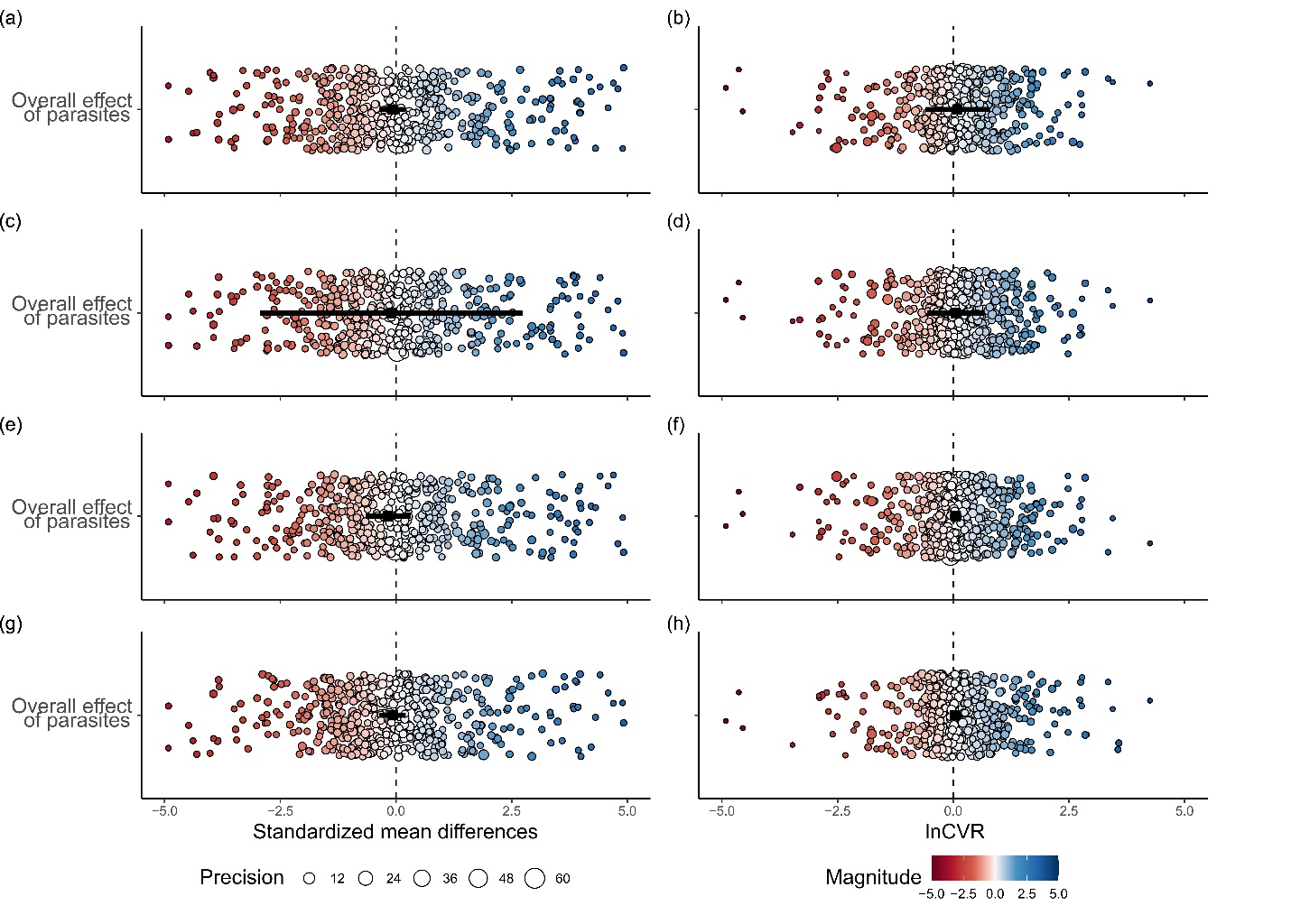
Figure S4** – Overall effects of parasites on species interaction mean responses (a, c, e, g) and response variance (b, d, f, h) when controlling for the effects of phylogeny and excluding viruses/unclassified parasites and including non-phylogenetic random effects for host and parasite species (a, b), when controlling for the effects of phylogeny and excluding viruses/unclassified parasites (c, d), not controlling for the effects of phylogeny and excluding viruses/unclassified parasites (e, f), and not controlling for the effects of phylogeny, including viruses/unclassified parasites, and excluding potentially influential outliers (g, h). (a), (c), (e), and (g) only show SMD effect sizes from -5 to 5 (Fig. S5 shows the full range). Negative SMD values represent a detrimental effect of parasites on hosts (i.e., reduced survival), while positive values represent an advantageous effect of parasites on hosts (i.e., increased survival). Large points in each panel denote estimated effects of parasites on mean fitness and fitness variation, respectively. Smaller points in each panel are individual effect sizes, color (hotter – more negative, cooler – more beneficial) denotes magnitude and sign of effect sizes, diameter denotes precision of estimates (1/SE), and error bars represent 95% CI’s.


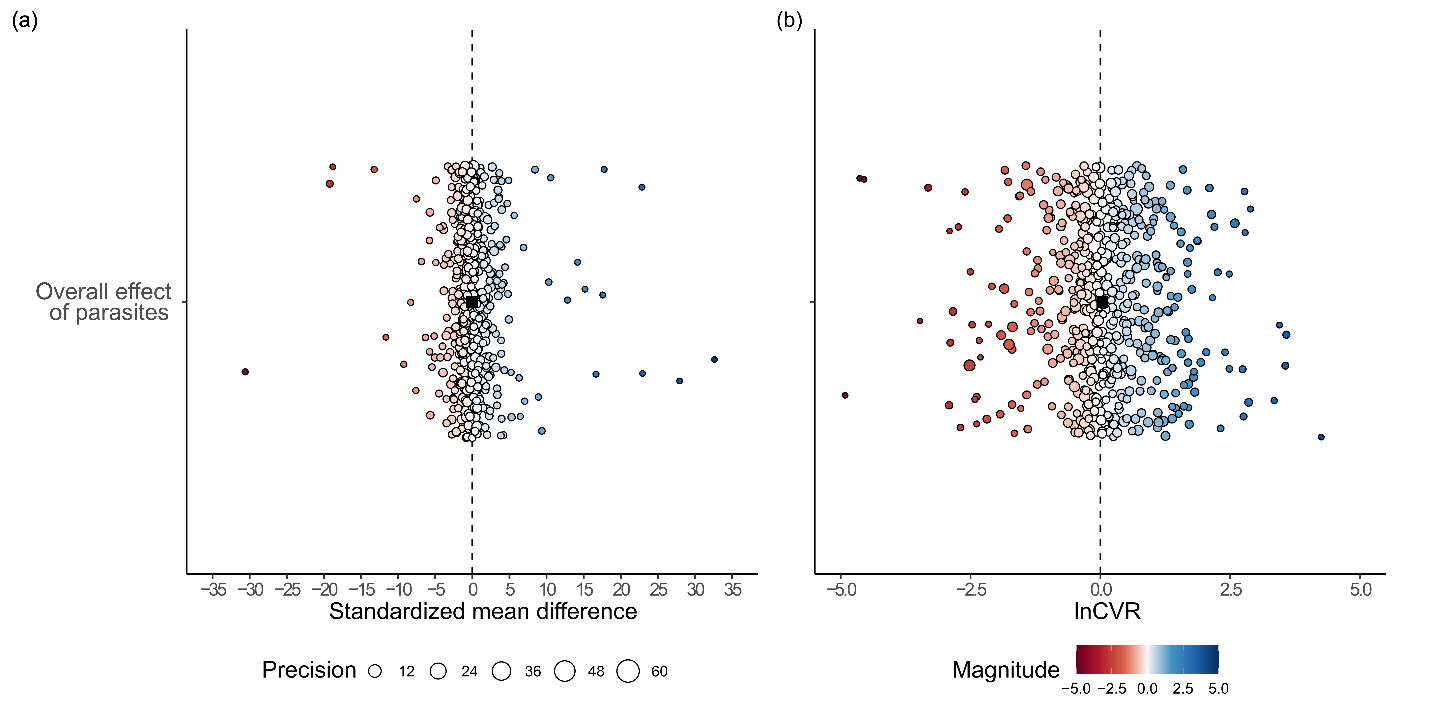


**Figure S5** – Overall effects of parasites on species interaction mean responses (a) and response variance (b) when not controlling for the effects of phylogeny and including viruses, showing the full range of effect sizes for mean responses. Negative SMD values represent a detrimental effect of parasites on hosts (i.e., reduced survival), while positive values represent an advantageous effect of parasites on hosts (i.e., increased survival). Large points in each panel denote estimated effects of parasites on mean fitness and fitness variation, respectively. Smaller points in each panel are individual effect sizes, color (hotter – more negative, cooler – more beneficial) denotes magnitude and sign of effect sizes, diameter denotes precision of estimates (1/SE), and error bars represent 95% CI’s.

**Table S1. –** Information on taxonomic assignments for the subset of host taxa assigned to higher taxonomic levels for inclusion in the phylogenetic analysis. Shown are the study, host taxa given in the study, and taxonomic assignment in the host phylogeny.

| Study | Host | Assigned taxa for phylogeny |
| --- | --- | --- |
| Avery et al 2020 | *Aleurothrixus trachoides* | *Aleurothrixus* |
| Bass & Weis 1999 | *Palaemonetes pugio* | *Palaemon pugio* |
| Benesh et al. 2008 | *Pallasea quadrispinosa* | *Pallaseopsis quadrispinosa* |
| Camp & Huizinga 1979 | *Asellus intermedius* | *Proasellus intermedius* |
| Dostál 2010 | *Conyza canadensis,*  *Stenactis annua* | *Erigeron canadensis,*  *Erigeron annuus* |
| Hechtel et al 1993 | *Caecidotea intermedius* | *Caecidotea intermedia* |
| Kok et al 1996 | *Carduus thoermeri* | *Carduss nutans* sbsp. *leiophyllus* |
| Krkošek et al 2011 | *Oncorhynchus gorbuscha* | *Oncorhynchus* |
| Mouttet et al 2011 | *Rosa hybrida* | *Rosa* |
| Parris & Beaudoin 2004 | *Hyla chrysoscelis* | *Dryophytes chrysoscelis* |
| Parris & Cornelius 2004 | *Bufo fowleri* | *Anazyrus fowleri* |
| Seppälä et al 2006 | *Oncorhynchus mykiss* | *Oncorhynchus* |
| Simon & Hilker 2005 | *Salix spp.* | *Salix* |
| Soghigian et al 2017 | *Aedes triseriatus* | *Ochlerotatus* |
| Washburn et al 1991 | *Aedes sierrensis* | *Ochlerotatus* |

**Table S2. –** Information on taxonomic assignments for the subset of parasite taxa assigned to higher taxonomic levels for inclusion in the phylogenetic analysis. Shown are the study, parasite taxa given in the study, and taxonomic assignment in the parasite phylogeny.

| Study | Parasite | Assigned taxa for phylogeny |
| --- | --- | --- |
| Aigbedion-Atalor et al 2021 | *Dolichogenidea gelechiidivoris* | *Apanteles gelechiidivoris* |
| Albornoz et al 2017 | *Phytophthora spp.* | *Phytophthora arenia* |
| Alma et al 2007 | *Paecilomyces fumosoroseus* | *Isaria fumosoroseus* |
| Altizer & Oberhauser 1995 | *Ophryocystis elektroscirrha* | Apicomplexa |
| Avery et al 2020 | *Cordyceps fumosorosea* | *Isaria fumosorosea* |
| Bacela-Spychalska et al 2014 | *Cucumispora dikogammari* | *Nosema* *dikogammari* |
| Belden & Wojdak 2011 | Platyhelminth spp. | *Echinostoma* |
| Benesh et al. 2008 | *Echinorhynchus borealis* | *Echinorhynchus* |
| Bunke et al 2015 | *Pleistophora mulleri* | *Pleistophora* |
| Campbell et al 2014 | Bacteria spp. | *Aquamarina spp.* |
| Cardoza et al 2002 | *Arachis hypgaea* | *Atherlia rolfsii* |
| Cipollini & Enright 2009 | *Erysiphe cruciferarum* | *Erysiphales* |
| Cipollini & Stiles 1993 | Fungi spp. | *Alternaria alternata* |
| Crabb & Pellmyr 2006 | *Digonogastra spp* | *Digonogastra* |
| Creissen et al 2016 | *Hyaloperonospora arabidopsidis* | *Halophytophthora* |
| de Azevedo et al 2017 | *Metarhizium brunneum* | *Metarhizium anisopliae* |
| Dick et al 2010 | *Echinorhynchus truttae* | *Echinorhynchus* |
| Dostál 2010 | Seed fungal pathogens | *Sclerotinia sclerotiorum* |
| Fielding et al 2003 | *Echinorhynchus truttae* | *Echinorhynchus* |
| Fischhoff et al 2018 | *Metarhizium brunneum* | *Metarhizium anisopliae* |
| Garcia et al. 2018 | *Oomycete spp* | *Aphanomyces ovidestruens* |
| Han et al. 2011 | *Batrachochytrium dendrobatidis* | Rhizophydiales |
| Herrick et al. 2008 | *Cotesia plutellae* | *Cotesia vestalis* |
| Kavaliers & Colwell 1995 | *Eimeria vermiformis* | Apicomplexa |
| Kendig et al 2021 | *Bipolaris gigantea* | *Drechslera gigantea* |
| Kiesecker & Blaustein 1999 | *Saprolegnia ferax* | *Saprolegnia* |
| Kok et al 1996 | *Puccinia carduorum* | *Puccinia calcitrapae* |
| Koprivnikar et al. 2008 | *Echinostoma trivolis* | *Echinostoma* |
| Kunz & Pung 2004 | *Colletotrichum turgidus* | *Microphallus turgidus* |
| Laverty et al 2017 | *Echinorhynchus truttae* | *Echinorhynchus* |
| Lefcort & Blaustein 1995 | *Candida humicola* | *Cryptococcus humicola* |
| MacNeil et al. 2003 | Microsporidian spp | *Pleistophora* |
| Marino et al. 2013 | *Echinostoma spp* | *Echinostoma* |
| Marino et al. 2014 | *Echinostoma spp* | *Echinostoma* |
| Marino et al. 2016 | *Echinostoma spp* | *Echinostoma* |
| Mathis & Tsutisui 2016 | *Pseudacteon lasciniosus, Pseudacteon planidorsalis* | *Pseudacteon* |
| Mottern et al. 2004 | *Pseudacteon tricuspis* | *Pseudacteon* |
| Parris & Beaudoin 2004 | *Batrachochytrium dendrobatidis* | Rhizophydiales |
| Parris & Cornelius 2004 | *Batrachochytrium dendrobatidis* | Rhizophydiales |
| Perrot-Minnot et al 2007 | *Pomphorhyncus tereticollis* | Platyhelminth |
| Preston & Sauer 2020 | *Echinostoma* *spp*., *Ribeiroira ondatrae, Echinostoma trivolvis, Alaria mustelae* | *Echinostoma* |
| Refardt & Ebert 2012 | Microsporidian spp | *Pleistophora* |
| Rosenkranz et al. 2018 | *Apatemon spp, Plagiorchioid spp* | *Apatemon*, Plagiorchioidea |
| Soh et al. 2013 | *Toxoplasma gondii* | Apicomplexa |
| Stephenson et al 2016 | *Gyrodactylus turnbulli* | *Gyrodactylus* |
| Tseng 2004 | *Ascogregarina taiwanensis* | Apicomplexa |
| Van Molken et al 2014 | *Albugo spp.* | *Albugo candida* |
| Voutilainen 2010 | *Plagiorchis elegans* | Plagiorchioidea |

**Section 2**

*Incorporating phylogeny*

One additional source of non-independence is the shared evolutionary histories of hosts and parasites (Chamberlain *et al.* 2012). Closely related hosts could exhibit similar responses to infection for a given interaction, or two closely related parasites could have similar effects on a host’s species interactions. To account for these sources of non-independence, we first constructed phylogenies of host and parasite species using the *rotl* package (Michonneau et al. 2016) to trim the Open Tree of Life (Hinchliff et al. 2015) such that it included only host (n = 123) or parasite taxa (n = 101). In one case (Slattery et al. 2013), we randomly assigned the hybrid host species used to calculate an effect size to one of its sister species, as the hybrid species was not defined on the Tree of Life. Many species were not well-resolved, thus, when necessary, they were collapsed into higher taxonomic levels (full list of host and parasite taxa collapsed into higher taxonomic levels in Table S1 and Table S2, respectively). All host and parasite taxa that were not defined at the species level or collapsed into higher taxonomic levels were assigned to genera. Polytomies in both the host and parasite trees were randomly resolved using the ape package (Paradis et al. 2004).

After constructing both phylogenies, we then constructed phylogenetic correlation matrices assuming full Brownian motion evolution (Lajeunesse 2009) using default settings for evolutionary rate parameters in the *ape* package (Paradis et al. 2004) and included them as random effects. Interactions between hosts and parasites are not likely to be dependent solely on either the host or the parasite, but instead relate to the shared evolutionary history of both organisms (Hadfield et al. 2014). We therefore also included a random effect of the interaction of the host and parasite phylogenies by calculating the tensor products of the correlation matrices (Lynch 1991; Hoeksema et al. 2018).

Additionally, simulation studies have suggested that decomposing species-level (both host and parasite) variance into phylogenetic random effects, as well as non-phylogenetic random effects (e.g., stemming from the same host or parasite species being used in multiple studies) is necessary to provide unbiased estimates of fixed effect in hierarchical meta-analyses (Cinar *et al.* 2021). To ensure that our models presented in the main text were robust to the potentially biased estimates that can result from not including these non-phylogenetic effects, we here included additional random effects for host and parasite species (Cinar *et al.* 2021), in addition to calculating the strength of the phylogenetic relationship (signal) among the host and parasite species. We estimated the latter by calculating the mean correlation of the phylogenetic correlation matrices.

We found that the mean phylogenetic signal among hosts and parasites was 0.331 and 0.330, respectively. Including host and parasite species as a random effects term in our models of the effects of parasites on overall host mean responses and response variance showed that there were no overall effects (*Ө_SMD_* = -0.02 [-2.86, 2.82], *Ө_lnCVR_* = 0.09 [-0.60, 0.78]. These results were not qualitatively different than our other models that did not include these terms (Fig. S4).

We further examined if the variance components for each random effect were identifiable using likelihood profile plots using the model controlling for phylogeny, excluding viruses, and including non-phylogenetic random effects of host and parasite species. For all full models of mean effects and response variances we found that the variance components of all random effects peaked at the same value as their parameter estimates from each respective model. Thus, the variance components were identifiable.

*Information on outlier analysis*

We examined for potentially influential outliers with Cook’s distance *d* (Cook 1977). Any effect size with values of *d* greater than three times the mean was considered an outlier (models of mean responses, with phylogeny: *d* > 0.0002, *n* = 19 effect sizes from 13 studies; without phylogeny: *d* > 0.002, *n* = 38 effect sizes from 29 studies; without phylogeny and with viruses: *d* > 0.002, *n* = 39 effect sizes from 30 studies; models of response variance, with phylogeny: *d* > 0.0002, *n* = 35 effect sizes from 21 studies; without phylogeny: *d* > 0.004, *n* = 36 effect sizes from 29 studies; without phylogeny and with viruses: *d* > 0.003, *n* = 41 effect sizes from 29 studies), and we ran our analyses again without these outliers. After removing outliers, the sample size for the analysis of mean responses with phylogeny had *n* = 489 effect sizes, without phylogeny had *n* = 469 effect sizes, and without phylogeny and with viruses had *n* = 543 effect sizes, while the sample size for the analysis of response variance with phylogeny had *n* = 459 effect sizes, without phylogeny had *n* = 459 effect sizes, and without phylogeny and with viruses had *n* = 524 effect sizes. The analysis of mean responses with phylogeny had a greater number of effects sizes than the analysis of mean responses without phylogeny because fewer of the effect sizes from the original analysis were identified as potential outliers when controlling for phylogeny.

*Further overview of studies in the database and overall effects of parasitism*

Forty-two effect sizes were extracted from 19 field-based studies, while 466 effect sizes were extracted from 119 laboratory studies. There was no significant difference between the effects of parasites in the lab or field for both mean responses and response variance between parasitized and non-parasitized groups for all three analyses (Wald-type tests of mean response model coefficients: *QM_phylogeny_* = 0.80, *p* = 0.37, *QM_without phylogeny_* = 0.31, *p* = 0.58, *QM_without phylogeny with viruses_* = 0.12, *p* = 0.73, Wald-type tests of response variance model coefficients: QM*_phylogeny_* = 0.19, *p* = 0.65, *QM_without phylogeny_* = 0.62, *p* = 0.43, *QM_without phylogeny with viruses_* = 1.70, *p* = 0.19).

Four hundred effect sizes were extracted from 95 studies that controlled parasitic infection within the study itself, while 108 effect sizes were extracted from 43 studies in which authors utilized previously infected hosts. There was no significant difference between the effects of parasites in the experimental studies or observational studies for both mean responses and response variance between controlled infections and previously infected hosts for all three analyses (Wald-type tests of mean response model coefficients: *QM_phylogeny_* = 0.27, *p* = 0.61, *QM_without phylogeny_* = 0.79, *p* = 0.35, *QM_without phylogeny with viruses_* = 1.38, *p* = 0.24, Wald-type tests of response variance model coefficients: *QM_phylogeny_* = 0.01, *p* = 0.92, *QM_without phylogeny_* = 0.81, *p* = 0.37, *QM_without phylogeny with viruses_* = 1.09, *p* = 0.29).

*List of papers in database*

1. Acharya, R., H.-S. Hwang, J.-K. Shim, Y.-S. Yu, and K.-Y. Lee. 2019. Control efficacy of fungus gnat, *Bradysia impatiens*, enhanced by a combination of entomopathogenic nematodes and predatory mites. Biological Control **138**.
2. Aeschlimann, P., M. Häberli, and M. Milinski. 2000. Threat-sensitive feeding strategy of immature sticklebacks (*Gasterosteus aculeatus*) in response to recent experimental infection with the cestode *Schistocephalus solidus*. Behavioral Ecology and Sociobiology **49**:1-7.
3. Agboton, B. V., R. Hanna, A. Onzo, S. Vidal, and A. von Tiedemann. 2013. Interactions between the predatory mite *Typhlodromalus aripo* and the entomopathogenic fungus *Neozygites tanajoae* and consequences for the suppression of their shared prey/host *Mononychellus tanajoa*. Experimental and Applied Acarology **60**:205-217.
4. Aigbedion-Atalor, P. O., M. P. Hill, P. M. Ayelo, S. Ndlela, M. P. Zalucki, and S. A. Mohamed. 2021. Can the combined use of the mirid predator *Nesidiocoris tenuis* and a braconid larval endoparasitoid *Dolichogenidea gelechiidivoris* improve the biological control of *Tuta absoluta*? Insects **12**:1004.
5. Albornoz, F. E., T. I. Burgess, H. Lambers, H. Etchells, and E. Laliberté. 2016. Native soilborne pathogens equalize differences in competitive ability between plants of contrasting nutrient‐acquisition strategies. Journal of Ecology **105**:549-557.
6. Alma, C. R., M. S. Goettel, B. D. Roitberg, and D. R. Gillespie. 2007. Combined effects of the entomopathogenic fungus, *Paecilomyces fumosoroseus* Apopka-97, and the generalist predator, *Dicyphus hesperus*, on whitefly populations. BioControl **52**:669-681.
7. Avery, P. B., V. Kumar, A. Francis, C. L. McKenzie, and L. S. Osborne. 2020. Compatibility of the predatory beetle, *Delphastus catalinae*, with an entomopathogenic fungus, *Cordyceps fumosorosea*, for biocontrol of invasive pepper whitefly, *Aleurothrixus trachoides*, in Florida. Insects **11**:590.
8. Bacela-Spychalska, K., T. Rigaud, and R. A. Wattier. 2014. A co-invasive microsporidian parasite that reduces the predatory behaviour of its host *Dikerogammarus villosus* (Crustacea, Amphipoda). Parasitology **141**:254-258.
9. Bakker, T. C. M., D. Mazzi, and S. Zala. 1997. Parasite-induced changes in behavior and color make *Gammarus pulex* more prone to fish predation. Ecology **78**:1098-1104.
10. Banerji, A., A. B. Duncan, J. S. Griffin, S. Humphries, O. L. Petchey, and O. Kaltz. 2015. Density‐ and trait‐mediated effects of a parasite and a predator in a tri‐trophic food web. Journal of Animal Ecology **84**:723-733.
11. Barahona, C. F. S., B. S. Threlkeld, P. B. Avery, A. W. Francis, and R. D. Cave. 2018. Compatibility and efficacy of the lady beetle *Thalassa montezumae* and the entomopathogenic fungus *Isaria fumosorosea* for biological control of the green croton scale: laboratory and greenhouse investigations. Arthropod-Plant Interactions **12**:715-723.
12. Barber, I., and G. D. Ruxton. 1998. Temporal prey distribution affects the competitive ability of  parasitized sticklebacks Animal Behaviour **56**:1477-1483.
13. Barney, J. N., A. DiTommaso, H. A. Baloch, and A. K. Watson. 2006. Fungal infection and soybean competition induce plastic responses in velvetleaf (*Abutilon theophrasti*) growth and reproductive output. Weed Science **54**:883-890.
14. Bass, C. S., and J. S. Weis. 1999. Behavioral changes in the grass shrimp, *Palaemonetes pugio* (Holthuis), induced by the parasitic isopod, *Probopyrus pandalicola* (Packard). Journal of Experimental Marine Biology and Ecology **241**:223-233.
15. Belden, L. K., and J. M. Wojdak. 2011. The combined influence of trematode parasites and predatory salamanders on wood frog (*Rana sylvatica*) tadpoles. Oecologia **166**:1077-1086.
16. Bell, H. A., R. E. Down, A. E. Kirkbride-Smith, and J. P. Edwards. 2004. Effect of microsporidian infection in *Lacanobia oleracea* (Lep., Noctuidae) on prey selection and consumption by the spined soldier bug *Podisus maculiventris* (Het., Pentatomidae). Journal of Applied Ecology **128**:548-553.
17. Benesh, D. P., J. Kitchen, K. Pulkkinen, I. Hakala, and E. T. Valtonen. 2008. The effect of *Echinorhynchus borealis* (Acanthocephala) infection on the anti-predator behavior of a benthic amphipod. Journal of Parasitology **94**:542-545.
18. Bernot, R. J., and G. A. Lamberti. 2008. Indirect effects of a parasite on a benthic community: an experiment with trematodes, snails and periphyton. Freshwater Biology **53**:322-329.
19. Bilu, E., and M. Coll. 2009. Parasitized aphids are inferior prey for a Coccinellid predator: implications for intraguild predation. Environmental Entomology **38**:153-158.
20. Boe, A., B. McDaniel, and K. Robbins. 1989. Direct effect of parasitism by D*inarmus acutus* Thomson on  seed predation by Canada milk-vetch Journal of Range Management **42**:514-515.
21. Brattey, J. 1983. The effects of larval Acanthocephalus lucii on the pigmentation, reproduction, and susceptibility to predation of the isopod Asellus aquaticus. The Journal of Parasitology **69**:1172-1173.
22. Brothers, C. A., and A. M. H. Blakeslee. 2021. Alien vs predator play hide and seek: how habitat complexity alters parasite mediated host survival. Journal of Experimental Marine Biology and Ecology **535**:151488.
23. Brown, A. F., and D. B. A. Thompson. 1986. Parasite manipulation of host behaviour: acanthocephalans and shrimps in the laboratory. Journal of Biologial Education **20**:121-127.
24. Bunke, M., M. E. Alexander, J. T. Dick, M. J. Hatcher, Paterson R., and A. M. Dunn. 2015. Eaten alive: cannibalism is enhanced by parasites. Royal Society Open Science **2**:140369.
25. Butler IV, M. J., J. M. Tiggelaar II, J. D. Shields, and M. J. Butler V. 2014. Effects of the parasitic dinoflagellate *Hematodinium perezi* on blue crab (*Callinectes sapidus*) behavior and predation. Journal of Experimental Marine Biology and Ecology **461**:381-388.
26. Calvo, F. J., J. D. Soriano, P. A. Stansly, and J. E. Belda. 2016. Can the parasitoid *Necremnus tutae* (Hymenoptera: Eulophidae) improve existing biological control of the tomato leafminer *Tuta aboluta* (Lepidoptera: Gelechiidae)? Bulletin of Entomological Research **106**:502-511.
27. Camp, J. W., and H. W. Huizinga. 1979. Altered color, behavior and predation susceptibility of the isopod *Asellus intermedius* infected with *Acanthocephalus dirus*. The Journal of Parasitology **65**:667-669.
28. Campbell, A. H., A. Vergés, and P. D. Steinberg. 2014. Demographic consequences of disease in a habitat‐forming seaweed and impacts on interactions between natural enemies. Ecology **95**:142-152.
29. Cardoza, Y. J., H. T. Alborn, and J. H. Tumlinson. 2002. *In vivo* volatile emissions from peanut plants induced by simultaneous fungal infection and insect damage. Journal of Chemical Ecology **28**:161-174.
30. Chailleux, A., P. Bearez, J. Pizzol, E. Amiens-Desneux, R. Ramirez-Romero, and N. Desneux. 2013. Potential for combined use of parasitoids and generalist predators for biological control of the key invasive tomato pest *Tuta absoluta*. Journal of Pest Science **86**:533-541.
31. Cipollini, D., and S. Enright. 2009. A powdery mildew fungus levels the playing field for garlic mustard (*Alliaria petiolata*) and a North American native plant. Invasive Plant Science and Management **2**:253-259.
32. Cipollini, M. L., and E. W. Stiles. 1993. Fruit rot, antifungal defense, and palatability of fleshy fruits for frugivorous birds. Ecology **74**:751-762.
33. Coors, A., and L. De Meester. 2008. Synergistic, antagonistic and additive effects of multiple stressors: predation threat, parasitism and pesticide exposure in *Daphnia magna*. Journal of Applied Ecology **45**:1820-1828.
34. Coslovsky, M., and H. Richner. 2012. An experimental test of predator–parasite interaction in a passerine bird. Oikos **121**:1691-1701.
35. Coyner, D. F., S. R. Schaack, M. G. Spalding, and D. J. Forrester. 2001. Altered predation susceptibility of mosquitofish infected with *Eustrongylides ignotus*. Journal of wildlife diseases **37**:556--560.
36. Crabb, B. A., and O. Pellmyr. 2006. Impact of the third trophic level in an obligate mutualism: do yucca plants benefit from parasitoids of yucca moths? International Journal of Plant Sciences **167**:119-124.
37. Creissen, H. E., T. H. Jorgensen, and J. K. M. Brown. 2016. Impact of disease on diversity and productivity of plant populations. Functional Ecology **30**:649-657.
38. Cuny, M. A. C., J. Gendry, J. Hernández-Cumplido, and B. Benrey. 2018. Changes in plant growth and seed production in wild lima bean in response to herbivory are attenuated by parasitoids. Oecologia **187**:447-457.
39. Currie, C. R. 2001. Prevalence and impact of a virulent parasite on a tripartite mutualism. Oecologia **128**:99-106.
40. Cáceres, C. E., C. J. Knight, and S. R. Hall. 2009. Predator–spreaders: predation can enhance parasite success in a planktonic host–parasite system. Ecology **90**:2850-2858.
41. Céspedes, V., M. I. Sánchez, and A. J. Green. 2017. Predator–prey interactions between native brine shrimp *Artemia parthenogenetica* and the alien boatman *Trichocorixa verticalis*: influence of salinity, predator sex, and size, abundance and parasitic status of prey. PeerJ **5**.
42. de Azevedo, A. G. C., B. M. Steinwender, J. Eilenberg, and L. Sigsgaard. 2017. Interactions among the predatory midge *Aphidoletes aphidimyza* (Diptera: Cecidomyiidae), the fungal pathogen *Metarhizium brunneum* (Ascomycota: Hypocreales), and maize-infesting aphids in greenhouse mesocosms. Insects **8**:44.
43. de Lange, E. S., K. Farnier, T. Degen, B. Gaudillat, R. Aguilar-Romero, F. Bahena-Juárez, K. Oyama, and T. C. J. Turlings. 2018. Parasitic wasps can reduce mortality of teosinte plants infested with fall armyworm: support for a defensive function of herbivore-induced plant volatiles. Frontiers in Ecology and Evolution **6**.
44. DeBlieux, T. S., and J. T. Hoverman. 2019. Parasite-induced vulnerability to predation in larval anurans. Diseases of Aquatic Organisms **135**:241-250.
45. Dianne, L., M.-J. Perrot-Minnot, A. Bauer, M. Gaillard, E. Léger, and T. Rigaud. 2011. Protection first then facilitation: a manipulative parasite modulates the vulnerability to predation of its intermediate host according to its own developmental stage. Evolution **65**:2692-2698.
46. Dick, J. T. A., M. Armstrong, H. C. Clarke, K. D. Farnsworth, M. J. Hatcher, M. Ennis, A. Kelly, and A. M. Dunn. 2010. Parasitism may enhance rather than reduce the predatory impact of an invader. Biology Letters **6**:636-638.
47. Ditommaso, A., and A. K. Watson. 1995. Impact of a fungal pathogen, *Colletotrichum coccodes* on growth and competitive ability of *Abutilon theophrasti*. The New Phytologist **131**:51-60.
48. Dostál, P. 2010. Post-dispersal seed mortality of exotic and native species: effects of fungal pathogens and seed predators. Basic and Applied Ecology **11**:676-684.
49. Dupont, C., A. Michiels, D. Sochard, Nathalie, S. Meyer, V. Brault, Y. Outreman, and A. Sentis. 2020. Virus mediated trophic interactions between aphids and their natural enemies. Oikos **129**:274-282.
50. Eberl, F., M. F. de Bobadilla, M. Reichelt, A. Hammerbacher, J. Gershenzon, and S. B. Unsicker. 2020. Herbivory meets fungivory: insect herbivores feed on plant pathogenic fungi for their own benefit. Ecology Letters **23**:1073-1084.
51. Ebssa, L., C. Borgmeister, and H.-M. Poehling. 2006. Simultaneous application of entomopathogenic nematodes and predatory mites to control western flower thrips *Frankliniella occidentalis*. Biological Control **39**:66-74.
52. Farahani, S., P. J. Palsbøll, I. Pen, and J. Komdeur. 2021. Effects of parasites upon non-host predator avoidance behaviour in native and invasive gammarids. Parasitology **148**:354-360.
53. Fielding, N. J., C. MacNeil, J. T. A. Dick, R. W. Elwood, G. E. Riddell, and A. M. Dunn. 2003. Effects of the acanthocephalan parasite *Echinorhynchus truttae* on the feeding ecology of *Gammarus pulex* (Crustacea: Amphipoda). Journal of Zoology **261**:321-325.
54. Fincham, W. N. W., A. M. Dunn, L. E. Brown, H. Hesketh, and H. E. Roy. 2019. Invasion success of a widespread invasive predator may be explained by a high predatory efficacy but may be influenced by pathogen infection. Biological Invasions **21**:3545-3560.
55. Fischhoff, I. R., J. C. Burtis, F. Keesing, and R. S. Ostfeld. 2018. Tritrophic interactions between a fungal pathogen, a spider predator, and the blacklegged tick. Ecology and Evolution **8**:7824-7834.
56. Fu, W., X. Yu, N. Ahmed, S. Zhang, and T. Liu. 2017. Intraguild predation on the aphid parasitoid *Aphelinus asychis* by the ladybird *Harmonia axyridis*. BioControl **62**:61-70.
57. Gabagambi, N. P., A.-G. V. Salvanes, F. Midtøy, and A. Skorping. 2019. The tapeworm *Ligula intestinalis* alters the behavior of the fish intermediate host *Engraulicypris sardella*, but only after it has become infective to the final host. Behavioural Processes **158**:47-52.
58. Gallagher, S. J., B. J. Tornabene, T. S. DeBlieux, K. M. Pochini, M. F. Chislock, Z. A. Compton, and L. K. Eiler, Verble, Kelton M. Hoverman, Jason T. 2019. Healthy but smaller herds: predators reduce pathogen transmission in an amphibian assemblage. Journal of Animal Ecology **88**:1613-1624.
59. Gao, F.-L., X.-X. Che, F.-H. Yu, and J.-M. Li. 2019. Cascading effects of nitrogen, rhizobia and parasitism via a host plant. Flora **251**:62-67.
60. Garcia, R. D., F. G. Jara, M. M. Steciow, and M. Reissig. 2018. Oomycete parasites in freshwater copepods of Patagonia: effects on survival and recruitment. Diseases of Aquatic Organisms **129**:123-134.
61. Grosholz, E. D. 1992. Interactions of intraspecific, interspecific, and apparent competition with host‐pathogen population dynamics. Ecology **73**:507-514.
62. Haislip, N. A., J. T. Hoverman, D. L. Miller, and M. J. Gray. 2012. Natural stressors and disease risk: does the threat of predation increase amphibian susceptibility to ranavirus? Canadian Journal of Zoology **90**:893-902.
63. Han, B. A., C. L. Searle, and B. A. R. 2011. Effects of an infectious fungus, *Batrachochytrium dendrobatidis*, on amphibian predator-prey interactions. PLoS ONE **6**.
64. Hechtel, L. J., C. L. Johnson, and S. A. Juliano. 1993. Modification of antipredator behavior of *Caecidotea intermedius* by its parasite *Acanthocephalus dirus*. Ecology **74**:710-713.
65. Herrick, N. J., S. R. Reitz, J. E. Carpenter, and C. W. O'Brien. 2008. Predation by *Podisus maculiventris* (Hemiptera: Pentatomidae) on *Plutella xylostella* (Lepidoptera: Plutellidae) larvae parasitized by *Cotesia plutellae* (Hymenoptera: Braconidae) and its impact on cabbage. Biological Control **45**:386-395.
66. Hyder, R., T. Pennanen, L. Hamberg, E. J. Vainio, T. Piri, and J. Hantula. 2013. Two viruses of *Heterobasidion* confer beneficial, cryptic or detrimental effects to their hosts in different situations. Fungal Ecology **6**:387-396.
67. Jacquin, L., Q. Mori, M. Pause, M. Steffen, and V. Medoc. 2014. Non-specific manipulation of gammarid behaviour by *P. minutus* parasite enhances their predation by definitive bird hosts. PLoS One **9**:e101684.
68. Kaldonski, N., M.-J. Perrot-Minnot, and F. Cézilly. 2007. Differential influence of two acanthocephalan parasites on the antipredator behaviour of their common intermediate host. Animal Behaviour **74**:1311-1317.
69. Kavaliers, M., and D. D. Colwell. 1995. Decreased predator avoidance in parasitized mice: neuromodulatory correlates. Parasitology **111**:257-263.
70. Kendig, A. E., V. J. Svahnström, A. Adhikari, P. F. Harmon, and S. L. Flory. 2021. Emerging fungal pathogen of an invasive grass: Implications for competition with native plant species. PLoS One **16**:e0237894.
71. Kennedy, C. R., P. F. Broughton, and P. M. Hine. 1978. The status of brown and rainbow trout, *Salmo trutta* and *S. gairdneri* as hosts of the acanthocephalan, *Pomphorhynchus laevis*. Journal of Fish Biology **13**:265-275.
72. Kiesecker, J. M., and A. R. Blaustein. 1999. Pathogen reverses competition between larval amphibians. Ecology **80**:2442-2448.
73. Kok, L. T., R. G. Abad, and A. B. A. M. Baudoin. 1996. Effects of *Puccinia carduorumon* musk thistle herbivores. Biological Control **6**:123-129.
74. Koprivnikar, J., M. R. Forbes, and R. L. Baker. 2008. Larval amphibian growth and development under varying density: are parasitized individuals poor competitors? Oecologia **155**:641-649.
75. Krkošek, M., J. S. Ford, A. Morton, S. Lele, R. A. Myers, and M. A. Lewis. 2007. Declining wild salmon populations in relation to parasites from farm salmon. Science **318**:1772-1775.
76. Kruess, A. 2002. Indirect interaction between a fungal plant pathogen and a herbivorous beetle of the weed *Cirsium arvense*. Oecologia **130**:563-569.
77. Kunz, A. K., and O. J. Pung. 2004. Effects of *Microphallus turgidus* (Trematoda: Microphallidae) on the predation, behavior, and swimming stamina of the grass shrimp *Palaemonetes pugio*. Journal of Parasitology **90**:441-445.
78. Lafferty, K. D., and A. K. Morris. 1996. Altered behavior of parasitized killifish increases susceptibility to predation by bird final hosts. Ecology **77**:1390-1397.
79. Lass, S., and K. Bittner. 2002. Facing multiple enemies: parasitised hosts respond to predator kairomones. Oecologia **132**:344-349.
80. Laverty, C., D. Brenner, C. McIlwaine, J. J. Lennon, J. T. A. Dick, F. E. Lucy, and K. A. Christian. 2017. Temperature rise and parasitic infection interact to increase the impact of an invasive species. International Journal for Parasitology **47**:291-296.
81. Lefcort, H., and A. R. Blaustein. 1995. Disease, predator avoidance, and vulnerability to predation in tadpoles. Oikos **74**:469-474.
82. Li, J., A. M. O. Oduor, Y. Feihai, and M. Dong. 2019. A native parasitic plant and soil microorganisms facilitate a native plant co‐occurrence with an invasive plant. Ecology and Evolution **9**:8652-8663.
83. Liere, H., and A. Larsen. 2010. Cascading trait‐mediation: disruption of a trait‐mediated mutualism by parasite‐induced behavioral modification. Oikos **199**:1394-1400.
84. Lin, L., T.-C. Shen, Y.-H. Chen, and S.-Y. Hwang. 2008. Responses of *Helicoverpa armigera* to tomato plants previously infected by ToMV or damaged by *H. armigera*. Journal of Chemical Ecology **34**:353-361.
85. Little, A. E., and C. R. Currie. 2009. Parasites may help stabilize cooperative relationships. BMC Evolutionary Biology **9**:1-9.
86. MacNeil, C., J. T. A. Dick, M. J. Hatcher, R. S. Terry, J. E. Smith, and A. M. Dunn. 2003. Parasite-mediated predation between native and invasive amphipods. Proceeding of the Royal Society B **270**:1309-1314.
87. Malek, J. C., and J. E. Byers. 2016. Predator effects on host-parasite interactions in the eastern oyster *Crassostrea virginica*. Marine Ecology Progress Series **556**:131-141.
88. Mansfield, S. 2019. Intraguild predation and prey preferences influence biological control of *Paropsis charybdis* by the southern ladybird, *Cleobora mellyi*. Biological Control **129**:164-170.
89. Marchetto, K. M., and A. G. Power. 2018. Coinfection timing drives host population dynamics through changes in virulence. The American Naturalist **191**:173-183.
90. Marino Jr., J. A., M. P. Holland, and M. J. Middlemis. 2014. Predators and trematode parasites jointly affect larval anuran functional traits and corticosterone levels. Oikos **123**:451-460.
91. Marino Jr., J. A., M. P. Holland, and E. E. Werner. 2016. Competition and host size mediate larval anuran interactions with trematode parasites. Freshwater Biology **61**:621-632.
92. Marino Jr., J. A., and E. E. Werner. 2013. Synergistic effects of predators and trematode parasites on larval green frog (*Rana clamitans*) survival. Ecology **94**:2697-2708.
93. Maronde, L., S. Losdat, and H. Richner. 2018. Do parasites and antioxidant availability affect begging behaviour, growth rate and resistance to oxidative stress? Journal of Evolutionary Biology **31**:904-913.
94. Marriott, D. R., M. L. Collins, R. M. Paris, D. R. Gudgin, C. J. Barnard, P. K. McGregor, F. S. Gilbert, J. C. Hartley, and J. M. Behnke. 1989. Behavioural modifications and increased predation risk of *Gammarus pulex* infected with *Polymorphus minutus*. Journal of Biologial Education **23**:135-141.
95. Mathis, K. A., and T. N. D. 2016. Dead ant walking: a myrmecophilous beetle predator uses parasitoid host location cues to selectively prey on parasitized ants. Proceedings of the Royal Society B: Biological Sciences **283**:20161281.
96. Milinski, M. 1985. Risk of predation of parasitized sticklebacks (G*asterosteus aculeatus* L.) under competition  for food Behaviour **93**:203-216.
97. Mirhosseini, M. A., Y. Fathipour, M. Soufbaf, and G. V. P. Reddy. 2019. Implications of using two natural enemies of *Tuta absoluta* (Lepidoptera: Gelechiidae) toward tomato yield enhancement. Bulletin of Entomological Research **109**:617-625.
98. Mohammed, A. A. 2018. *Lecanicillium muscarium* and *Adalia bipunctata* combination for the control of black bean aphid, *Aphis fabae*. BioControl **63**:277-287.
99. Moore, J. 1983. Responses of an avian predator and its isopod prey to an acanthocephalan parasite. Ecology **64**:1000-1015.
100. Moral, R. d. A., C. G. B. Demétrio, J. Hinde, W. A. C. Godoy, and F. S. Fernandes. 2017. Parasitism-mediated prey selectivity in laboratory conditions and implications for biological control. Basic and Applied Ecology **19**:67-75.
101. Mottaghinia, L., M. Hassanpour, J. Razmjou, E. Chamani, and M. Hosseini. 2018. Intraguild predation on the parasitoid wasp *Aphidius colemani* by the predator *Aphidoletes aphidimyza*: effect of host plant cultivars. Journal of Agricultural Science and Technology **20**:533-542.
102. Mottern, J. L., K. M. Heinz, and P. J. Ode. 2004. Evaluating biological control of fire ants using phorid flies: effects on competitive interactions. Biological Control **30**:566-583.
103. Mouttet, R., P. Bearez, C. Thomas, and N. Desneux. 2011. Phytophagous arthropods and a pathogen sharing a host plant: evidence for indirect plant-mediated interactions. PLoS One **6**:e18840.
104. Niemelä, M., A. Markkola, and P. Mutikainen. 2008. Modification of competition between two grass species by a hemiparasitic plant and simulated grazing. Basic and Applied Ecology **9**:117-125.
105. Nunes, G. d. S., C. C. Truzi, J. do Nascimento, F. F. de Paula, S. T. S. de Matos, R. A. Polanczyk, and S. A. De Bortoli. 2019a. *Beauveria bassiana* (Ascomycota: Hypocreales)–treated diamondback moth (Lepidoptera: Plutellidae) larvae mediate the preference and functional response of *Euborellia annulipes* (Dermaptera: Anisolabididae) nymphs. Journal of Economic Entomology **112**:2614-2619.
106. Nunes, G. S., D. G. Ramalho, N. A. dos Santos, C. C. Truzi, N. F. Vieira, C. P. Cardoso, and S. A. De Bortoli. 2019b. Parasitism-mediated interactions between the ring-legged earwig and sugarcane borer larvae. Neotropical Entomology **48**:919-926.
107. Ondračková, M., M. Dávidová, M. Gelnar, and P. Jurajda. 2006. Susceptibility of Prussian carp infected by metacercariae of *Posthodiplostomum cuticola* (v. Nordmann, 1832) to fish predation. Ecological Research **21**:526-529.
108. Onzo, A., I. A. Bello, and R. Hanna. 2013. Effects of the entomopathogenic fungus *Neozygites tanajoae* and the predatory mite *Typhlodromalus aripo* on cassava green mite densities: screenhouse experiments. BioControl **58**:397-405.
109. Otieno, J. A., P. Pallmann, and H.-M. Poehling. 2016. Additive and synergistic interactions amongst *Orius laevigatus* (Heteroptera: Anthocoridae), entomopathogens and azadirachtin for controlling western flower thrips (Thysanoptera: Thripidae). BioControl **62**:85-95.
110. Overholt, W. A., L. Markle, E. Rosskopf, V. Manrique, J. Albano, E. Cave, and S. Adkins. 2009. The interactions of tropical soda apple mosaic tobamovirus and *Gratiana boliviana* (Coleoptera: Chrysomelidae), an introduced biological control agent of tropical soda apple (*Solanum viarum*). Biological Control **48**:294-300.
111. Parris, M. J., and J. G. Beaudoin. 2004. Chytridiomycosis impacts predator-prey interactions in larval amphibian communities. Oecologia **140**:626-632.
112. Parris, M. J., and T. O. Cornelius. 2004. Fungal pathogen causes competitive and developmental stress in larval amphibian communities. Ecology **85**:3385-3395.
113. Parris, M. J., A. Davis, and J. P. Collins. 2004. Single-host pathogen effects on mortality and behavioral responses to predators in salamanders (Urodela: Ambystomatidae). Canadian Journal of Zoology **82**:1477-1483.
114. Parris, M. J., A. Storfer, J. P. Collins, and E. W. Davidson. 2005. Life-history responses to pathogens in tiger salamander (*Ambystoma tigrinum*) larvae. Journal of Herpetology **39**:366-372.
115. Penczykowski, R. M., M. S. Shocket, J. Housley Ochs, B. C. P. Lemanski, H. Sundar, m. M. A. Duffy, and S. R. Hall. 2022. Virulent disease epidemics can increase host density by depressing foraging of hosts*. The American Naturalist **199**:75-90.
116. Perrot-Minnot, M.-J., N. Kaldonski, and F. Cézilly. 2007. Increased susceptibility to predation and altered anti-predator behaviour in an acanthocephalan-infected amphipod. International Journal for Parasitology **37**:645-651.
117. Peñaflor, M. F. G. V., K. E. Mauck, K. J. Alves, C. M. de Moraes, and M. C. Mescher. 2016. Effects of single and mixed infections of Bean pod mottle virus and Soybean mosaic virus on host‐plant chemistry and host–vector interactions. Functional Ecology **30**:1648-1659.
118. Pourian, H.-R., R. Talaei-Hassanloui, A. A. Kosari, and A. Ashouri. 2011. Effects of *Metarhizium anisopliae* on searching, feeding and predation by *Orius albidipennis* (Hem., Anthocoridae) on *Thrips tabaci* (Thy., Thripidae) larvae. Biocontrol Science and Technology **21**:15-21.
119. Preston, D. L., and E. L. Sauer. 2020. Infection pathology and competition mediate host biomass overcompensation from disease. Ecology **101**:e03000.
120. Pérez-Jvostov, F., A. P. Hendry, G. F. Fussmann, and M. E. Scott. 2016. An experimental test of antagonistic effects of competition and parasitism on host performance in semi‐natural mesocosms. Oikos **125**:790-796.
121. Rayamajhi, M. B., P. D. Pratt, T. D. Center, and T. K. Van. 2010. Insects and a pathogen suppress *Melaleuca quinquenervia* cut-stump regrowth in Florida. Biological Control **53**:1-8.
122. Rayamajhi, M. B., T. K. Van, P. D. Pratt, and T. D. Center. 2006. Interactive association between *Puccinia psidii* and *Oxyops vitiosa*, two introduced natural enemies of *Melaleuca quinquenervia* in Florida. Biological Control **37**:56-67.
123. Refardt, D., and D. Ebert. 2012. The impact of infection on host competition and its relationship to parasite persistence in a *Daphnia* microparasite system. Evolutionary Ecology **26**:95-107.
124. Reigada, C., S. B. L. Araujo, M. A. M. de Aguiar, J. Z. Gião, P. R. Guimarães Jr, L. A. Trinca, and W. A. C. Godoy. 2013. Impacts of enemy‐mediated effects and the additivity of interactions in an insect trophic system. Population Ecology **55**:11-26.
125. Ridenour, W. L., and R. M. Callaway. 2003. Root herbivores, pathogenic fungi, and competition between *Centaurea maculosa* and *Festuca idahoensis*. Plant Ecology **169**:161-170.
126. Rosenkranz, M., R. Poulin, and C. Selbach. 2018. Behavioural impacts of trematodes on their snail host: Species‐specific effects or generalised response? Ethology **124**:790-795.
127. Sacco, L. H., C. P. Goater, T.-D. Smith, D. P. Chivers, and M. C. O. Ferrari. 2021. Escape responses to simulated host versus nonhost predators in minnows exposed to a brain-encysting parasite. Animal Behaviour **173**:169-176.
128. Seppälä, O., A. Karvonen, M. Kuosa, M. Haataja, and J. Jokela. 2013. Are sick individuals weak competitors? Competitive ability of snails parasitized by a gigantism-inducing trematode. Plos One **8**:e79366.
129. Seppälä, O., A. Karvonen, and E. T. Valtonen. 2006. Susceptibility of eye fluke-infected fish to predation by bird hosts. Parasitology **132**:575-579.
130. Seppälä, O., E. T. Valtonen, and D. P. Benesh. 2008. Host manipulation by parasites in the world of dead-end predators: adaptation to enhance transmission? Proceedings of the Royal Society B **275**:1611-1645.
131. Shirakashi, S., K. Teruya, and K. Ogawa. 2008. Altered behaviour and reduced survival of juvenile olive flounder, *Paralichthys olivaceus*, infected by an invasive monogenean, *Neoheterobothrium hirame*. International Journal for Parasitology **38**:1513-1522.
132. Simelane, D. O., D. C. Steinkraus, and T. J. Kring. 2008. Predation rate and development of *Coccinella septempunctata* L. influenced by *Neozygites fresenii*-infected cotton aphid prey. Biological Control **44**:128-135.
133. Simon, M., and M. Hilker. 2005. Does rust infection of willow affect feeding and oviposition behavior of willow leaf beetles? Journal of Insect Behavior **18**:115-129.
134. Sisterson, M. S., and A. L. Averill. 2003. Interactions between parasitized and unparasitized conspecifics: parasitoids modulate competitive dynamics. Oecologia **135**:362-371.
135. Slattery, M., D. A. Renegar, and D. J. Gochfeld. 2013. Direct and indirect effects of a new disease of alcyonacean soft corals. Coral Reefs **32**:879-889.
136. Soghigian, J., L. R. Valsdottir, and T. P. Livdahl. 2017. A parasite's modification of host behavior reduces predation on its host. Ecology and Evolution **7**:1453-1461.
137. Soh, L. J. T., A. Vasudevan, and A. Vyas. 2013. Infection with *Toxoplasma gondii* does not elicit predator aversion in male mice nor increase their attractiveness in terms of mate choice. Parasitology Research:3373-3378.
138. Stafford-Banks, C. A., L. H. Yang, M. S. McMunn, and D. E. Ullman. 2014. Virus infection alters the predatory behavior of an omnivorous vector. Oikos **123**:1384-1390.
139. Steen, H., M. Taitt, and C. J. Krebs. 2002. Risk of parasite-induced predation: an experimental field study on Townsend's voles (*Microtus townsendii*). Canadian Journal of Zoology **80**:1286-1292.
140. Stephenson, J. F., C. Kinsella, J. Cable, and C. van Oosterhout. 2016. A further cost for the sicker sex? Evidence for male-biased parasite-induced vulnerability to predation. Ecology and Evolution **6**:2506-2515.
141. Sun, Z., Z. Liu, W. Zhou, H. Jin, H. Liu, A. Zhou, A. Zhang, and M.-Q. Wang. 2016. Temporal interactions of plant - insect - predator after infection of bacterial pathogen on rice plants. Scientific Reports **6**:1-12.
142. Swartz, S. J., G. A. De Leo, C. L. Wood, and S. H. Sokolow. 2015. Infection with schistosome parasites in snails leads to increased predation by prawns: implications for human schistosomiasis control.
143. Toscano, B. J., B. Newsome, and B. D. Griffen. 2014. Parasite modification of predator functional response. Oecologia **175**:345-352.
144. Tseng, M. 2004. Sex-specific response of a mosquito to parasites and crowding. Proceeding of the Royal Society B **271**:S186-S188.
145. Turner, P. J., L. Morin, D. G. Williams, and D. J. Kriticos. 2010. Interactions between a leafhopper and rust fungus on the invasive plant *Asparagus asparagoides* in Australia: a case of two agents being better than one for biological control. Biological Control **54**:322-330.
146. Vallon, M., N. Anthes, and K. U. Heubel. 2016. Water mold infection but not paternity induces selective filial cannibalism in a goby. Ecology and Evolution **6**:7221-7229.
147. van Molken, T., H. de Caluwe, C. A. Hordijk, A. Leon-Reyes, T. A. L. Snoeren, N. M. van Dam, and J. F. Stuefer. 2012. Virus infection decreases the attractiveness of white clover plants for a non-vectoring herbivore. Oecologia **170**:433-444.
148. van Mölken, T., V. Kuzina, K. R. Munk, C. E. Olsen, T. Sundelin, N. M. van Dam, and T. P. Hauser. 2014. Consequences of combined herbivore feeding and pathogen infection for fitness of *Barbarea vulgaris* plants. Oecologia **175**:589-600.
149. Voutilainen, A. 2010. Interactive effects of predation risk and parasitism on the circadian rhythm of foraging activity in the great pond snail Lymnaea stagnalis (Gastropoda: Lymnaeidae). Annales de Limnologie - International Journal of Limnology **46**:217-223.
150. Washburn, J. O., D. R. Mercer, and J. R. Anderson. 1991. Regulatory role of parasites: impact on host population shifts with resource availability. Science **253**:185-188.
151. Wedekind, C., and M. Milinski. 1996. Do three-spined sticklebacks avoid consuming copepods, the first intermediate host of *Schistocephalus solidus*? — an experimental analysis of behavioural resistance. Parasitology **112**:371-383.
152. Wu, S., Z. Xing, W. Sun, X. Xu, R. Meng, and Z. Leia. 2018. Effects of *Beauveria bassiana* on predation and behavior of the predatory mite *Phytoseiulus persimilis*. Journal of Invertebrate Pathology **153**:51-56.
153. Xue, Y., C. A. Bahlai, A. Frewin, C. M. McCreary, L. E. Des Marteaux, A. W. Schaafsma, and R. H. Hallett. 2012. Intraguild predation of the aphid parasitoid *Aphelinus certus* by *Coccinella septempunctata* and *Harmonia axyridis*. BioControl **57**:627-634.
154. Yan, G., L. Stevens, C. J. Goodnight, and J. J. Schall. 1998. Effects of a tapeworm parasite on the competition of *Tribolium* beetles. Ecology **79**:1093-1103.
